# A novel *in vitro* system for simultaneous infections with hepatitis B, C, D and E viruses

**DOI:** 10.1016/j.jhepr.2025.101383

**Published:** 2025-02-28

**Authors:** Roxanne Fouillé, Eloi R. Verrier, Amse De Meyer, Lieven Verhoye, Maud Michelet, Romain Barnault, Caroline Pons, Olivier Diaz, Michel Rivoire, Guillaume Passot, Eike Steinmann, Heiner Wedemeyer, Anna Salvetti, Nicole Pavio, Virginie Doceul, Raphaël Darteil, Philip Meuleman, David Durantel, Julie Lucifora

**Affiliations:** 1 CIRI, Centre International de Recherche en Infectiologie, Univ Lyon, Inserm, U1111, Université Claude Bernard Lyon 1, CNRS, UMR5308, ENS de Lyon, F-69007, Lyon, France; 2 Université de Strasbourg, Inserm, Institut de Recherche sur les Maladies Virales et Hépatiques UMR_S1110, Strasbourg, France; 3 Laboratory of Liver Infectious Diseases, Department of Diagnostic Sciences, Faculty of Medicine and Health Sciences, Ghent University, Ghent, Belgium; 4 INSERM, U1052, Cancer Research Center of Lyon (CRCL), University of Lyon (UCBL1), CNRS UMR_5286, Centre Léon Bérard, Lyon, France; 5 Centre Léon Bérard (CLB), INSERM, U1032, Lyon, France; 6 Service de chirurgie générale et oncologique, Hôpital Lyon Sud, Hospices Civils de Lyon Et CICLY, EA3738, université Lyon 1, France; 7 German Centre for Infection Research (DZIF), Department for Molecular & Medical Virology, Ruhr University Bochum, 44801 Bochum, Germany; 8 Dept. of Gastroenterology, Hepatology, Infectious DIseases and Endocrinology, Hannover Medical School, Hannover, Germany; 9 Agence Nationale de Sécurité Sanitaire de L'alimentation de L'environnement et du Travail (ANSES), Institut National de Recherche pour L'agriculture L'alimentation et L'environnement (INRAE), École Nationale Vétérinaire d'Alfort (ENVA), UMR Virology, 94700 Maisons-Alfort, France; 10 ENYO Pharma, Lyon, France; 11 German Centre for Infection Research (DZIF), partner-site Hannover-Braunschweig, Excellence Cluster RESIST, D-SOLVE consortium, Germany

**Keywords:** Hepatitis B Virus, Hepatitis D Virus, Hepatitis C Virus, Hepatitis E Virus, hepatocytes, drug screening, broad acting antivirals

## Abstract

**Background & Aims:**

The liver, and more precisely hepatocytes, can be infected by several hepatotropic viruses, including HBV, HDV, HCV and HEV, with chronic infection leading to end-stage liver diseases. Since no *in vitro* model allowing multi-infections with the four viruses is reported, limited data are available on their interplay as well as on the potential cross-reactivity of antivirals in multi-infection cases. The aim of our study was to set up such a model.

**Methods:**

HuH7.5-NTCP cells were cultured with 2% DMSO (dimethyl sulfoxide) for 1 week to allow partial differentiation into hepatocytes (dHuH7.5-NTCP) before infection with the different viruses and treatment with known antiviral molecules.

**Results:**

We observed increased expression of liver specific transcripts and production of ApoB containing VLDL in dHuH7.5-NTCP cells and replication of HBV, HDV, HCV and HEV for at least 4 weeks after mono or multiple infections. We recapitulated the known antiviral effect of sofosbuvir on HCV and HEV (>90% reduction in the levels of intracellular viral RNAs, *p* <0.0005) and of IFN-α on HCV, HEV and HDV (80% reduction in the levels of intracellular viral RNAs, *p* <0.0005). Besides its already described antiviral effect on HBV and HDV, we observed that GW4064, a farnesoid X receptor (FXR) agonist, also strongly inhibited HEV replication (85 to 95% reduction in the levels of intracellular HEV RNAs, *p* <0.0005). Using HEV-infected HuHep mice, we confirmed the antiviral effect of vonafexor, an FXR agonist, that is currently being tested clinically against HBV/HDV.

**Conclusions:**

We set-up the first *in vitro* model allowing multi-infections with hepatitis viruses that can be used for broad drug screening and highlighted FXR ligands as potential broad-acting antivirals.

**Impact and implications:**

Hepatitis virus infections caused by HBV, HCV, HDV, and HEV represent a global health threat. Treatment options remain limited, notably due to the lack of knowledge about molecular virus-host interactions. Moreover, the interplay between these four viruses in the context of co-infections remains unknown. In this study, we report the first *in vitro* system that allows for mono and multi-infections with these four viruses and characterize the broad antiviral activity of farnesoid X receptor agonists, paving the way for the development of new strategies for viral cure.

## Highlights

• HuH7.5-NTCP cells can be partially differentiated into hepatocyte-like cells using DMSO.
• dHuH7.5-NTCP allow replication of HBV, HDV, HCV and HEV for at least 4 weeks after mono or multiple infections.
• Treatment with FXR agonists leads to a reduction of HBV, HDV and HEV infection.

## Graphical abstract

**Figure:**
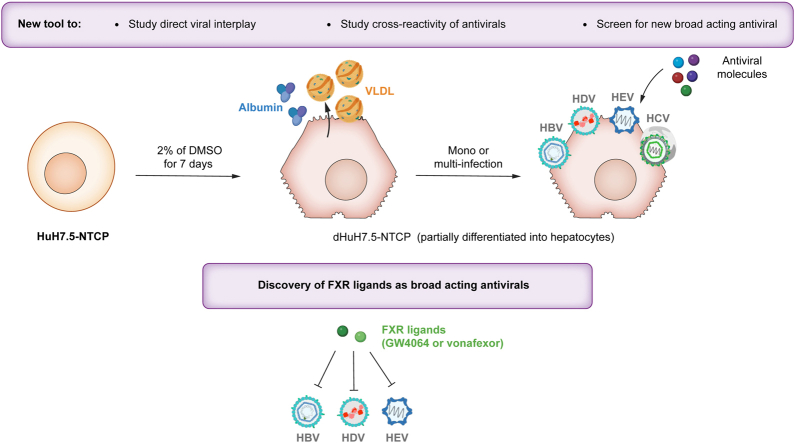

## Introduction

The liver, and in particular hepatocytes, are the target of several pathogens, including viruses such as HBV, HCV, HDV and HEV, which may impair their essential functions and trigger liver inflammation, fibrosis, cirrhosis, hepatocellular carcinoma and eventually lead to liver failure.^1^ If HCV infection can be cured with specific direct-acting antivirals (DAAs), chronic HBV and HDV infections can only be contained using either nucleos(t)ide analogues or bulevirtide, but without viral clearance.^1^ Ribavirin or, in some specific cases, pegylated–IFN–α, are used in clinic against chronic hepatitis E, but failures are often reported.^1^^,^^2^ Very few epidemiological studies are available concerning multi-infections with several hepatitis viruses. Apart from the well-known HBV/HDV co-infections, infections with double or triple viruses have been reported[3], [4], [5], [6] and these cases, as well as quadruple infections, are probably underestimated due to deficient diagnosis, in particular for HEV and HDV. Importantly, multi-infections with hepatitis viruses increase the risk of fulminant hepatitis,^7^ and severe liver diseases.^8^^,^^9^ Although HBV, HCV, HDV and HEV infections are restricted to hepatocytes, very few data are available concerning their potential interplay. Beside the well documented interference of HDV on HBV,^10^^,^^11^ conflicting data have been reported on the interactions between other hepatitis viruses. It has been suggested that HCV may dominate HBV, HDV and HEV *in vivo*,^12^^,^^13^ but HBV and HCV were found to replicate in the same cell without interference in HuH7 cells^14^ and a case report described HCV clearance during acute HBV/HDV super-infection.^15^ HEV was once found associated with repressed HBV replication,^16^ whereas a case report described an asymptomatic HEV superinfection followed by a flare in HBV replication in an HBsAg carrier without signs of HBV replication for 8 years.^17^ It is therefore not clear if and how one hepatitis virus would negatively interfere with or boost the replication of other hepatitis viruses in case of multi-infections *in vivo*. Moreover, the effects of the current antiviral treatments on co- or multi-infections are unknown and concerns about viral reactivation after clearance of one infection among the others have been raised. Finally, new antiviral strategies against HBV, HDV and HEV are needed, including broadly acting therapies that would ease the management of multi-infected patients. In this context, it is urgent to develop a system allowing researchers to determine how antiviral drugs may influence hepatitis viruses that are not initially targeted by the treatment in case of multiple infections.

HBV, HCV, HDV and HEV naturally infect and replicate into highly differentiated, non-dividing, human hepatocytes. Therefore, we aimed to establish a relevant and scalable *in vitro* hepatocyte culture model supporting the replication of these four viruses. Although primary human hepatocytes (PHHs) are the gold standard for HBV and HDV *in vitro* studies,^18^ few data have been reported on their susceptibility to HEV infection,^19^ and permissiveness to HCV is contested. Moreover, the use of PHHs is problematic, due to inter-individual variability and difficult access. Similarly, differentiated HepaRG (dHepaRG) cells are susceptible to HBV, HDV, and HEV infections^20^^,^^21^ but not to HCV infection. HuH7 cells and its derivate clone, HuH7.5, are susceptible to HCV and HEV^22^^,^^23^ but do not express the HBV/HDV receptor NTCP.^24^ Interestingly, HuH7 cells treated with dimethyl sulfoxide (DMSO) display a more differentiated phenotype.^25^

We therefore investigated whether a 1-week treatment of HuH7-NTCP and HuH7.5-NTCP cell cultures with 2% DMSO may lead to their differentiation into hepatocyte like-cells and support the infection by these four major hepatitis viruses.

## Materials and methods

The materials and methods used are described in the supplementary information.

## Results

**Fig. 1:**
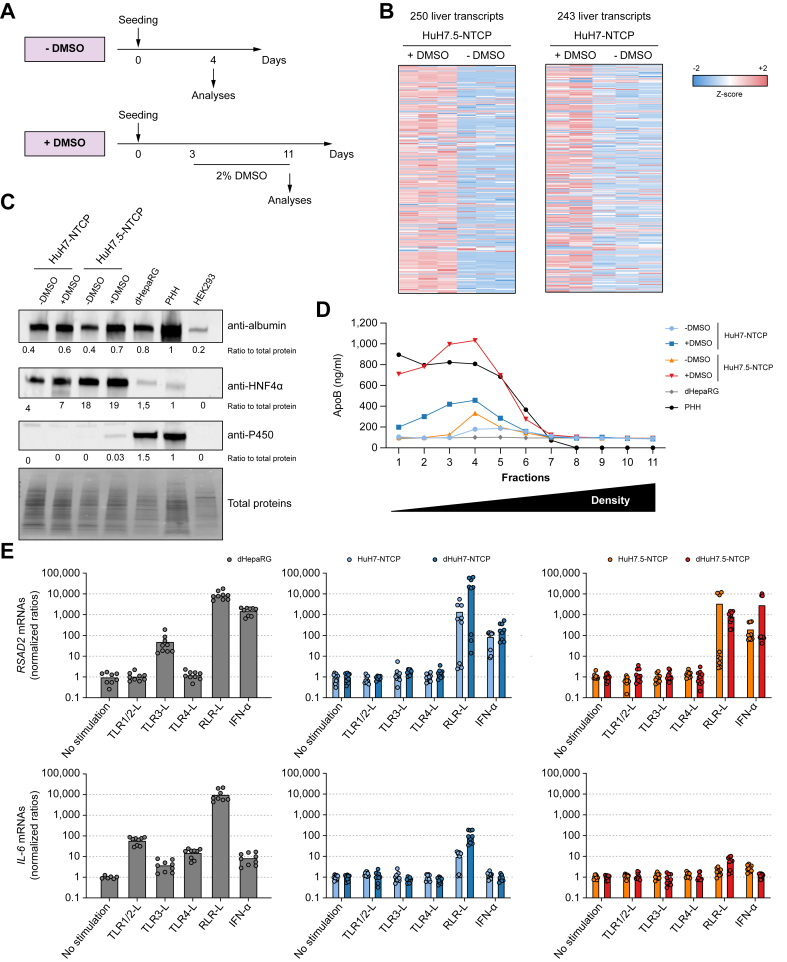
DMSO treatment of HuH7.5-NTCP cells enables their partial differentiation into hepatocyte-like cells. (A-E) HuH7-NTCP or HuH7.5-NTCP cells were seeded and treated or not with 2% DMSO. (B) Cells were lysed, total RNAs were extracted and gene expression was assessed by RNA sequencing (GSE288204). Relative liver-specific gene expression (Z-score) of up to 250 genes (see Table S1 for details) is presented. (C, D, E) HepaRG cells were differentiated for 4 weeks (dHepaRG) and PHHs seeded for 24 h before analyses. (C) Cells were lysed and levels of the indicated proteins were assessed by western blot. Results from a representative experiment are presented. (D) 24 h before analysis, media were replaced by serum-free media. Supernatants were collected and ApoB was assessed by ELISA in each fraction of iodixanol gradients. Results from a representative experiment are presented. (E) Cells were stimulated or not with the indicated molecules for 24 h. Total RNAs were extracted, and gene expression was assessed by reverse-transcription quantitative PCR. Levels of target mRNAs were normalized to the levels of Gus-B mRNAs and the no stimulation conditions. Data are the mean of three independent experiments each performed with three biological replicates. dHepaRG, differentiated HepaRG; PHHs, primary human hepatocytes.

Through transcriptomic analyses in both cell lines, we identified DMSO-induced upregulated genes, mainly belonging to liver-specific pathways such as drug metabolism or primary bile acid biosynthesis (Fig. S1A). Analyses of relative liver gene expression according to the Human Protein Atlas showed that DMSO-treated HuH7-NTCP (dHuH7-NTCP) and HuH7.5-NTCP (dHuH7.5-NTCP) cells expressed most of the liver-specific genes (Fig. 1A,B, Table S2). In contrast, genes related to cell cycle or DNA replication pathways were downregulated upon treatment of cells with DMSO (Fig. S1A). Western blot and quantitative reverse-transcription PCR analyses indicated that, like PHHs and dHepaRG cells, dHuH7.5-NTCP cells expressed albumin, a blood protein secreted by differentiated hepatocytes, and HNF4α, an hepatocyte-specific transcription factor, essential for HBV RNA synthesis,^26^ indicating the differentiation of cells. However, dHuH7.5-NTCP cells produced lower levels of CYP34A/P450 proteins than PHHs or dHepaRG cells (Fig. 1C and S1B). Additional transcriptomic analyses confirmed lower levels of several cytochrome transcripts in dHuH7.5-NTCP cells compared to dHepaRG cells (Fig. S2A). These data suggest that dHuH7.5-NTCP cells are less efficient in drug detoxification than PHHs and dHepaRG cells, a property that may help to unravel potential toxicity issues of antiviral molecules in drug screening assays. One of the key functions of hepatocytes is to release very low-density lipoprotein (VLDL) containing non-exchangeable ApoB into the bloodstream to supply body tissues with triglycerides. We observed that both dHuH7-NTCP and dHuH7.5-NTCP cells produced higher amounts of triglycerides in comparison to non-differentiated cells (Fig. 1D). Despite identical ApoB secretion before differentiation of these two cell lines, only dHuH7.5-NTCP cells were able to produce comparable levels of ApoB-containing VLDL to PHHs (Fig. 1D), thus suggesting an advantage of this subclone over the parental HuH7 cell line. Of note dHepaRG cells did not produce a measurable amount of ApoB-containing VLDL (Fig. 1D) and HuH7.5-NTCP cells displayed higher levels of most apolipoprotein transcripts compared to dHepaRG cells (Fig. S2B). An innate immune response to pathogens and especially pattern recognition receptor activation is also a strong feature of differentiated hepatocytes^27^ and numerous immune pathways are impaired in hepatoma cell lines.^28^ We therefore stimulated HuH7-NTCP and HuH7.5-NTCP cells with several pattern recognition receptor ligands to determine if they can recover some innate functions upon DMSO treatment (Fig. 1E). We confirmed the increased levels of *RSAD2* and *IL-6* transcripts upon TLR1/2, TLR3, TLR4, RLR ligands and IFN-α stimulations of dHepaRG cells.^28^ In contrast, even after treatment with DMSO of HuH7-NTCP and HuH7.5-NTCP cells, levels of *RSAD2* transcripts were only increased following stimulations with RLR ligand and IFN-α, but not following TLR1/2, TLR3 or TLR4 stimulations. These data are in accordance with the levels of *TLR1, TLR2, TLR3* and *TLR4* transcripts being at least 1 log lower at steady state in dHuH7.5-NTCP cells compared to dHepaRG cells (Fig. S2C). Of note, compared to dHepaRG cells, the induction of *IL-6* transcripts was modest in HuH7-NTCP cells stimulated with an RLR ligand and very weak in HuH7.5-NTCP cells, irrespective of their differentiation status (Fig. 1E). The default of IL-6 production by dHuH7.5-NTCP cells might be due to lower levels of the *TRAF5* transcripts, required for NF-kB activation,^29^ in dHuH7.5-NTCP cells compared to dHepaRG cells at steady state (Fig. S2C). Altogether, our data indicate that treatment with DMSO allows for partial differentiation of HuH7-NTCP and HuH7.5-NTCP cells into hepatocyte-like cells with dHuH7.5-NTCP cells having a stronger hepatocyte phenotype than dHuH7-NTCP cells.

**Fig. 2:**
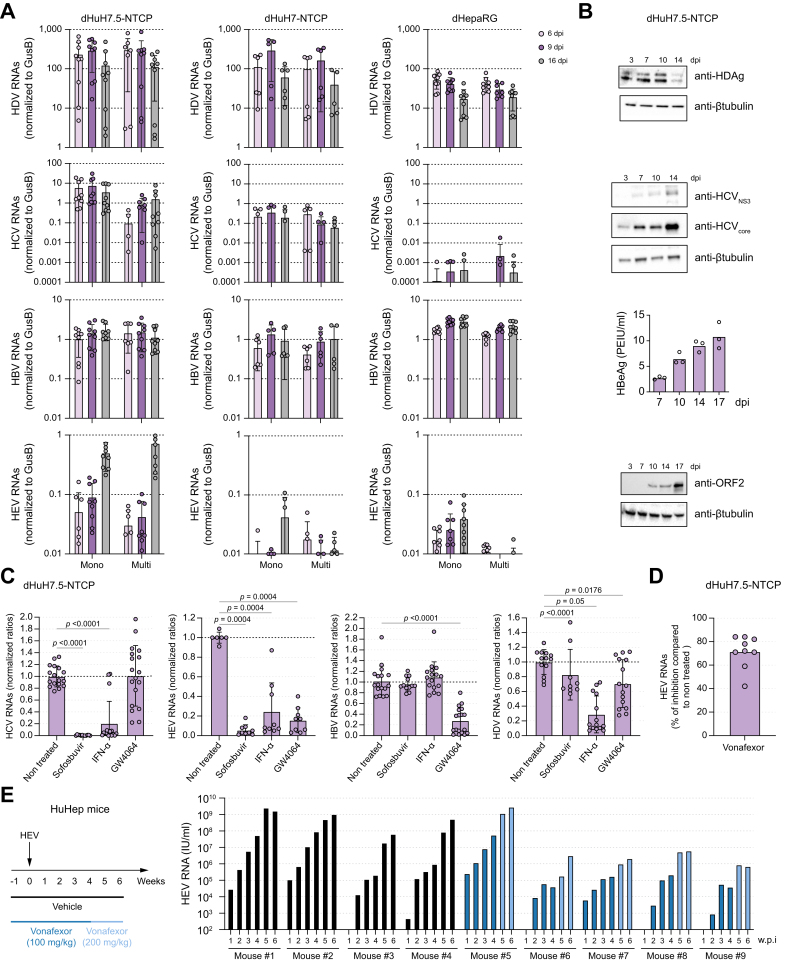
Infections of dHuH7.5-NTCP cells by HBV, HCV, HDV and HEV and testing for broadly acting antivirals. (A) dHuH7-NTCP, dHuH7.5-NTCP, or dHepaRG cells were inoculated with HBV, HCV, HDV, or HEV (mono) or with the four viruses at the same time (multi). At the indicated dpi, cells were lysed and the levels of viral RNAs were assessed by multiplex RT-ddPCR. (B) dHuH7.5-NTCP cells were inoculated with HCV, HDV, HEV, or HBV. At the indicated dpi, cells were lysed and levels of intracellular viral proteins were analyzed by western blot and the levels of HBeAg in the cell supernatants were quantified by CLIA. (C, D) dHuH7.5-NTCP cells were inoculated with HBV, HCV, HDV, or HEV. Three dpi with HBV, HCV, HDV or 10 dpi with HEV, cells were treated or not with the indicated molecules for 10 days. Cells were lysed and the levels of intracellular viral RNAs were assessed by RT-qPCR. Data are the mean ± SD of at least three independent experiments each performed with three biological replicates. (E) HuHep mice were treated as indicated. At the indicated weeks post-infection, levels of HEV RNA were quantified by qRT-PCR in 10% (w/v) stool suspensions of the infected mice. CLIA, chemiluminescence immunoassay; dpi, days post-inoculation; dHepaRG, differentiated HepaRG; dHuH7-NTCP, DMSO-treated HuH7-NTCP; dHuH7.5-NTCP, DMSO-treated HuH7.5-NTCP; qRT-PCR, quantitative reverse-transcription PCR; RT-ddPCR, reverse-transcription droplet digital PCR. Statistical analyses were performed using a Mann-Whitney U test with the prism software.

To analyze multi-infections with HBV, HCV, HDV and HEV, we developed a multiplex RT-ddPCR (reverse-transcription droplet digital PCR) assay that allows for the specific and absolute quantification of each of the viral RNAs in a single assay (Fig. S3). Differentiated HuH7-NTCP, dHuH7.5-NTCP, and dHepaRG cells were inoculated with HBV, HDV, HCV, HEV or with the four viruses simultaneously, and levels of intracellular viral RNAs were assessed by multiplex RT-ddPCR at different time points post-inoculation (Fig. 2A). HDV inoculation led to strong replication in the three different cell lines in both mono- or multi-infection settings with a usual peak of replication around 6-9 days post-inoculation. HCV inoculation also led to potent replication in dHuH7-NTCP and dHuH7.5-NTCP cells, but not in dHepaRG cells in both mono- or multi-infection settings. It should be noted that multi-infection of dHuH7.5-NTCP cells limited HCV replication at day 6 post inoculation, suggesting a possible negative interference of the other viruses on HCV replication at an early time point. HBV inoculation led to comparable HBV RNAs levels in all the tested conditions and cell types. Within the time frame of the experiment, production of HEV RNAs were only observed in dHuH7.5-NTCP cells at day 16 post-inoculation with quasi-enveloped HEV particles (Fig. 2A). These data suggested that only dHuH7.5-NTCP cells can support an efficient replication of the four viruses in mono-infection or co-infection settings. Additional experiments at later time points confirmed persistent replication of HBV, HCV, HDV and HEV in dHuH7.5-NTCP cells in mono-infection (Fig. S4). We also detected viral antigen production by western blot and CLIA (chemiluminescence immunoassay) analyses after mono-infection of dHuH7.5-NTCP cells (Fig. 2B). Of note, double or triple combinations of infection with HBV, HCV, HDV and HEV could also be performed in dHuH7.5-NTCP cells (Fig. S3B) and the interferon-independent viral interference of HDV on HBV we already described^11^ could be recapitulated (Fig. S3C). Altogether, our data indicated that dHuH7.5-NTCP cells support mono- and simultaneous multi-infections with HBV, HCV, HDV and HEV and it is likely that dHuH7.5-NTCP cells may also allow for super-infections. However, it remains to be determined if the four viruses can replicate in the same cell or whether exclusion/interference mechanisms exist as suggested for HCV (Fig. 2A). Future single-cell analyses may help to resolve this important question.

To determine if dHuH7.5-NTCP cells are suitable to screen for broadly acting antiviral molecules, we tested three known antivirals in mono-infection settings (Fig. 2C). As expected, sofosbuvir, a nucleoside analogue used to treat HCV-infected patients^30^ abrogated HCV infection in dHuH7.5-NTCP cells and highly reduced the levels of HEV RNAs^2^ (>90% reduction in the levels of intracellular viral RNAs, *p* <0.0005), without affecting HBV and HDV RNA levels. Moreover, IFN-α treatment following virus inoculation strongly decreased the levels of intracellular HCV, HDV and HEV RNAs (80% reduction in the levels of intracellular viral RNAs, *p* <0.0005). However, in contrast to what is usually observed in HBV-infected cells,^31^^,^^32^ IFN-α had no effect on the levels of HBV RNAs in dHuH7.5-NTCP cells. We recently reported that FXR (farnesoid X receptor) ligands, such as GW4064, can inhibit HBV and HDV replication *in vitro*.^32^ Herein, we confirmed this result in dHuH7.5-NTCP cells, with no effect on HCV RNA levels. Interestingly, GW4064 strongly decreased the levels of HEV RNAs by up to 85% (*p* <0.0001) in dHuH7.5-NTCP cells infected with cell culture-derived quasi-enveloped HEV (Fig. 2C) and vonafexor, a clinical candidate currently being tested against HBV and soon against HDV, showed a similar antiviral effect on HEV (Fig. 2D). We confirmed the strong anti-HEV effect of GW4064 using naked particles of HEV P6 Kernow strain to infect dHuH7.5-NTCP cells (>95% reduction in the levels of intracellular viral RNAs, *p* = 0.0022) (Fig. S5). Finally, we confirmed that treatment of HuHep mice with vonafexor attenuated HEV infection in four treated animals out of five (Fig. 2E). After cessation of therapy, all four infected HuHep mice experienced a rapid increase in fecal HEV RNA (Fig. S6) to the plateaued levels reached by the control non-treated mice.

## Discussion

Altogether, we showed here that two hepatocyte-like cell lines, each with its own advantages and disadvantages, can be used to overcome some issues encountered when working with PHHs. While the immortalized dHepaRG cells exhibit a much more accurate profile regarding innate immune and detoxification pathways, their ability to replicate HCV and HEV is limited. In contrast, the transformed dHuH7.5-NTCP cells display impaired innate immune pathways, but likely a more relevant functional hepatocyte-like lipid metabolism and allow for efficient (multi)-infections with HBV, HDV, HCV and HEV. Of note, even if dHuH7.5-NTCP cells will not permit the investigation of innate immune response to viral infection and its role regarding potential viral interference in the case of multi-infections, their differing innate responsiveness compared to dHepaRG cells or PHHs could be a useful tool. For instance, treatment of dHuH7.5-NTCP cells with IFN-α did not result in a reduction in the levels of intracellular HBV RNAs, in contrast to what is usually observed in HBV-infected HepaRG cells and PHHs.^31^^,^^32^ We excluded a default in the JAK/STAT pathway in dHuH7.5-NTCP since IFN-α treatment reduced the levels of intracellular HCV, HDV and HEV RNAs (Fig. 2C) and induced IFN-stimulated gene (ISG) expression (Fig. 1E). We instead hypothesize that a different set of ISGs might be produced in dHuH7.5-NTCP cells upon IFN-α treatment, which could explain the differential response. Identification of anti-HBV ISGs could therefore be undertaken by differential analysis.

In conclusion, we established a unique *in vitro* hepatocyte culture model supporting simultaneous infection with the four main hepatotropic viruses (HBV, HCV, HDV and HEV) and allowing us to (i) study the direct interplay between those viruses, (ii) investigate the potential cross-reactivity of antivirals in the case of multi-infections and (iii) screen for/investigate broadly acting antivirals. Additionally, our data highlights FXR ligands, particularly the clinical stage candidate vonafexor, as potential broad-acting antivirals against at least three hepatotropic viruses. Further studies in preclinical models are warranted to move forward in clinical trials.

## Abbreviations

DMSO, dimethyl sulfoxide; dHepaRG, differentiated HepaRG; dHuH7-NTCP, DMSO-treated HuH7-NTCP; dHuH7.5-NTCP, DMSO-treated HuH7.5-NTCP; ISG, IFN-stimulated gene; PHHs, primary human hepatocytes; VLDL, very low-density lipoprotein.

## Financial support

This work was supported by several grants from the 10.13039/501100003323ANRS MIE (French national agency for research on AIDS, viral hepatitis and emerging diseases, CSS12, ECTZ172540, ANRS0544, ECTZ187893, ECTZ244976), as well as financial support of INSERM and 10.13039/501100004794CNRS. RF was supported by PhD scholarships from University Claude Bernard Lyon 1. E.R.V. acknowledges fundings from the 10.13039/501100001665French National Research Agency (ANR, grant number ANR-21-CE15-0035-01 DELTArget) and fundings from the interdisciplinary Thematic Institute IMCBio, as part of the ITI 2021-2028 program of the 10.13039/501100003768University of Strasbourg, 10.13039/501100004794CNRS and 10.13039/501100001677Inserm, was supported by IdEx Unistra (ANR-10-IDEX-0002), and by SFRI-STRAT’US project (ANR-20-SFRI-0012) and 10.13039/501100001828EUR IMCBio (ANR-17-EURE-0023) under the framework of the French Investments for the Future Program. 10.13039/501100001670ES was supported by a grant of the German Centre for Infection Diseases (10.13039/100009139DZIF). PM was supported by 10.13039/501100004385Ghent University (PhD fellowship to 10.13039/100010238ADM, and 10.13039/501100007229BOF.BAF.2024.0637.01) and grants from the Research Foundation-10.13039/501100011878Flanders (FWO-Vlaanderen; Excellence of Science (EOS) project VirEOS2.0 and research project G0A7Y24N).

## Authors’ contributions

Study concept and design: JL, DD, PM; Acquisition of data: RF, MM, CP, ADM, LV; Analyses and interpretation of data: RF, EV, PM, DD, JL; Drafting of the manuscript: RF, EV, DD, JL; Funding acquisition: JL, DD, AS, PM; Material support: OD, RB, VD, NP, ES, GP, MR, RD, PM, HW.

## Data availability statement

The data presented in this manuscript are available through the corresponding authors (Julie Lucifora and David Durantel) upon reasonable request. Next-generation sequencing was performed by the Biomedical Sequencing Facility at CeMM Research Center for Molecular Medicine of the Austrian Academy of Sciences. RNA-seq data presented in this manuscript are accessible through Gene Expression Omnibus (GSE288203 and GEO GSE288204).

## Conflict of interest

The authors of this study declare that they do not have any conflict of interest.

Please refer to the accompanying ICMJE disclosure forms for further details.
