# Supplementary material for: A novel in vitro system for simultaneous infections with hepatitis B, C, D and E viruses
Source: JHEP Rep. 2025 Feb 28;7(5):101383. doi: 10.1016/j.jhepr.2025.101383 (PMC11999259; doi:10.1016/j.jhepr.2025.101383)
Supplement: Multimedia component 1 [file mmc1.pdf]

A novel *in vitro* system for simultaneous infections with hepatitis B, C, D and E viruses

Roxanne Fouillé, Eloi R. Verrier, Amse De Meyer, Lieven Verhoye, Maud Michelet, Romain Barnault, Caroline Pons, Olivier Diaz, Michel Rivoire, Guillaume Passot, Eike Steinmann, Heiner Wedemeyer, Anna Salvetti, Nicole Pavio, Virginie Doceul, Raphaël Darteil, Philip Meuleman, David Durantel, Julie Lucifora

Table of contents

Materials and methods……………………………………………………………………2

Supplementary figures……………………………………………………………………5

Original western blots……………………………………………………………………11

Supplementary tables……………………………………………………………..…………12

Supplementary references…………………………………………………………………...18

Materials and methods

Cell culture and viral infection. HepaRG cells were cultured and differentiated as previously described [1, 2]. Primary human hepatocytes (PHH) were isolated from human liver resections obtained from the Centre Léon Bérard (Lyon) and Hopital de Lyon Sud with French ministerial authorizations (AC 2013-1871, DC 2013 – 1870, AFNOR NF 96 900 sept 2011) as previously described [3]. The HuH7-NTCP and HuH7.5-NTCP are cultured in 10% FCS-supplemented DMEM and differentiated with 2% of DMSO for 1 week without splitting the cells. HBV (genotype D) inocula were prepared from HepAD38 supernatants [4] and used with a multiplicity of infection (m.o.i) of 1000 vge/cells. HDV inocula (genotype 1) were prepared from supernatants from HuH7-2C8D as previously described [5] and used with a m.o.i of 500 vge/cells. Viral stocks of HCV genotype 2A JFH1 strain were generated as previously described [6] and used with a m.o.i of 0,2 vge/cells. Quasi-enveloped HEV-3 was produced by infection of dHepaRG cells and collection of supernatant as previously described [7] and used with a m.o.i of 10 vge/cells. Alternatively, intracellular cell culture HEV-3 p6 Kernow (naked virus) was also used in Figure S4 and S5 [8]. Infections were performed overnight with 4% PEG.

Reagents. TLR1/2 ligand (Pam3CSK4, used at 10 ug/mL), TRL3-L (Riboxxol, used at 10 ug/mL), TLR4-L (LPS, used at 10 ug/mL), RLR-L (transfected polyI:C, used at 1 ug/mL) or IFN-α (used at 500 or 1000 IU/mL). Pam3CSK4, LPS and PolyIC HMW were purchased from Invivogen. Riboxxol was purchased from Ribox and IFN-α (Roferon, used at 500 IU/mL) was purchased from Roche. Sofosbuvir (used at 10 µM) and GW4064 (used at 10 µM) were purchased from Selleckchem, RG7834 (used at 10 µM) was synthetized by AI-Biopharma. Vonafexor, provided by ENYO Pharma, was used at 10 µM.

Protein expression analysis. For western blot analyses, cells were lyzed with a RIPA lysis buffer (Tris-HCl pH 7,5 10mM, NaCl 140mM, EDTA 1mM, EGTA 0,5mM, 1% Triton X100, 0,1% SDS, 0,1% Na-Deoxycholate) containing protease and phosphatase inhibitors (Roche). We used the following primary antibodies: anti-γ-tubulin (Sigma, T6557), anti-albumin (Dako), anti-ORF2 (Milipore MAB8002), anti-NS3 (Virogen 217-A), anti-HCV core (Thermofisher MA1-7366). The anti-HDAg, a polyclonal rabbit antibody was produced by Eurogentec for in-house use. HBsAg were detected in the supernatant of HBV-infected cells using the Autobio kit according to the manufacturer (AutoBio, China)

ApoB secretion and VLDL analyses. To assess ApoB containing VLDL production, cell supernatants were loaded on a 7-56 % iodixanol gradient as previously described [9]. After an overnight ultracentrifugation, fractions were collected and levels of ApoB were quantified by ELISA in each fraction using anti-ApoB (Biodesign H45640M) and anti-ApoB conjugated with HRP (Biodesign K34005G).

Nucleic acids detection. Total intracellular RNAs were extracted with the “Monarch, nucleic acid purification kit” according to the manufacturer’s instructions (New England Biolabs). RNA reverse transcription assays were performed using the LunaScript RT Super Mix (New England BioLabs). Quantitative PCR was performed using specific primers (Table 1) and normalized to the household gene Gus-B using the Luna Universal qPCR Master Mix (New England BioLabs). To detect multi-infections, we set up a multiplex RT-ddPCR assay using the “ddPCR Multiplex Supermix” (Bio-Rad) according to the manufacturer’s instruction with specific primers (0,9 uM final) and probes (0,25 uM final) (Table S1). Droplets were generated using and the” QX200™ Droplet Generator” (Bio-Rad) and analyzed after PCR with the “QX600 Droplet Reader” (Bio-Rad).

HuHep mice experiment. Human-liver chimeric mice (HuHep mice) were generated by transplanting 10^6 primary human hepatocytes (Lonza, Basel, Switzerland; donor HUM191501) into the spleen of uPA$^{+/+}$-SCID mice. Successful humanization was confirmed by quantification of human albumin in mouse plasma using a human-specific ELISA (Bethyl Laboratories, USA). Mice with human albumin levels ranging between 3-16 mg/mL were used in this study. For *in vivo* prevention of HEV infection,

HuHep mice (n=6) received a 7-week Vonafexor treatment starting 7 days before HEV inoculation (intraperitoneal injection of a filtered stool suspension containing 10^4 IU of a mouse-passaged genotype 3 patient isolate). Vonafexor was formulated as a suspension (15 and 25 mg/ml) in deionized water supplemented with 0.5% carboxymethylcellulose and 0.25% Tween80, and was administered daily via oral gavage. Vonafexor was dosed at 100 mg/kg (15 mg/ml) until 4 weeks post-infection, after which the dose was increased to 200 mg/kg (25 mg/ml). Two weeks later treatment was stopped. Control animals were treated with vehicle only, according to the same regimen as Vonafexor. Stool samples were collected weekly and HEV RNA content was analyzed in 10% (w/v) suspensions as described before [10]. Briefly, total RNA was extracted from a 10% (w/v) stool suspension with the NucliSENS easyMAG device according to the manufacturer's instructions (Biomérieux). Quantitative PCR was performed on the LightCycler 480 (Roche Diagnostics) by using the LightCycler Multiplex RNA Virus Master mix (Roche) and specific primers (5'-GGTGGTTTCTGGGGTGAC-3' and 3'-AGGGGTTGGTTGGATGAA-5') and probe (5'-FAM-TGATTCTCAGCCCTTCGC-TAMRA-3'). Quantification was performed by using an in-house standard curve based on the WHO 1st international standard of HEV (Paul-Ehrlich Institute). All mice were bred under sterile conditions and all experiments were approved by the Animal Ethics Committee of the Faculty of Medicine and Health Sciences of Ghent University (ref. ECD 22/46)

Statistical analyses. Statistical were performed using a Mann-Whitney U test with the prism software. p values are indicated directly in the graphs.

Next-Generation Sequencing and Raw Data Acquisition. Two RNA-seq datasets were produced and are accessible through Gene Expression Omnibus (GSE288204 and GSE288203). Expression profiling libraries were prepared using NEBNext Ultra II Directional RNA with UMI Adaptors kit (New England Biolabs) and Stranded mRNA kit (Illumina) for GEO DIF and GEO IFNa, respectively. Libraries were sequenced on a HiSeq 3000 (GSE288204) or a HiSeq 4000 (GSE288203) instrument (Illumina, San Diego, CA, USA) following a 50-base-pair, single-end recipe. Raw data acquisition (HiSeq Control Software, HCS, HD 3.4.0.38) and base calling (Real-Time Analysis Software, RTA, 2.7.7) was performed on-instrument, while the subsequent raw data processing off the instruments involved two custom programs based on Picard tools (2.19.2). In a first step, base calls were converted into lane-specific, multiplexed, una-ligned BAM files suitable for long-term archival (IlluminaBasecallsToMultiplexSam, 2.19.2-CeMM). In a second step, archive BAM files were demultiplexed into sample-specific, unaligned BAM files (Illumi-naSamDemux, 2.19.2-CeMM).

Transcriptome Analysis. NGS reads were mapped to the Genome Reference Consortium GRCh38 assembly via "Spliced Transcripts Alignment to a Reference" (STAR, 2.7.5a) utilizing the "basic" ensembl transcript annotation from version e100 (April 2020) as reference transcriptome. Since the hg38 assembly flavour of the UCSC Genome Browser was preferred for downstream data processing with Bioconductor packages for entirely technical reasons, ensembl transcript annotation had to be adjusted to UCSC Genome Browser sequence region names. STAR was run with options recommended by the ENCODE project. NGS read alignments overlapping ensembl transcript features were counted with the Bioconductor (3.12 and 3.11) Genomic Alignments (1.26.0 and 1.24.0) package (GSE288204 and GSE288203, respectively). Transcript-level counts were aggregated to gene-level counts and the Bioconductor DESeq2 (1.30.0 for GEO DIF and 1.28.1 for GSE288203) package was used to test for differential expression based on a model using the negative binomial distribution. Pathway analysis was performed through Gene Set Enrichment Analysis (GSEA) [11]. Regarding GSE288204, replicate number 2 of differentiated Huh7-NTCP cells was considered as an outlier and removed from the analysis. The expression of 263 liver-specific genes according the Human Protein Atlas [12] was investigated (https://www.proteinatlas.org/humanproteome/tissue/liver#the_liver_specific_proteome, accessed online on November 4, 2024). Excluding the non-expressed genes in the cell lines, the expression of

250 and 243 genes are presented for HuH7.5-NTCP and HuH7-NTCP cells (Figure 1B and Table S2), respectively, using the Z-score transformation as already published [13].

Supplementary figures

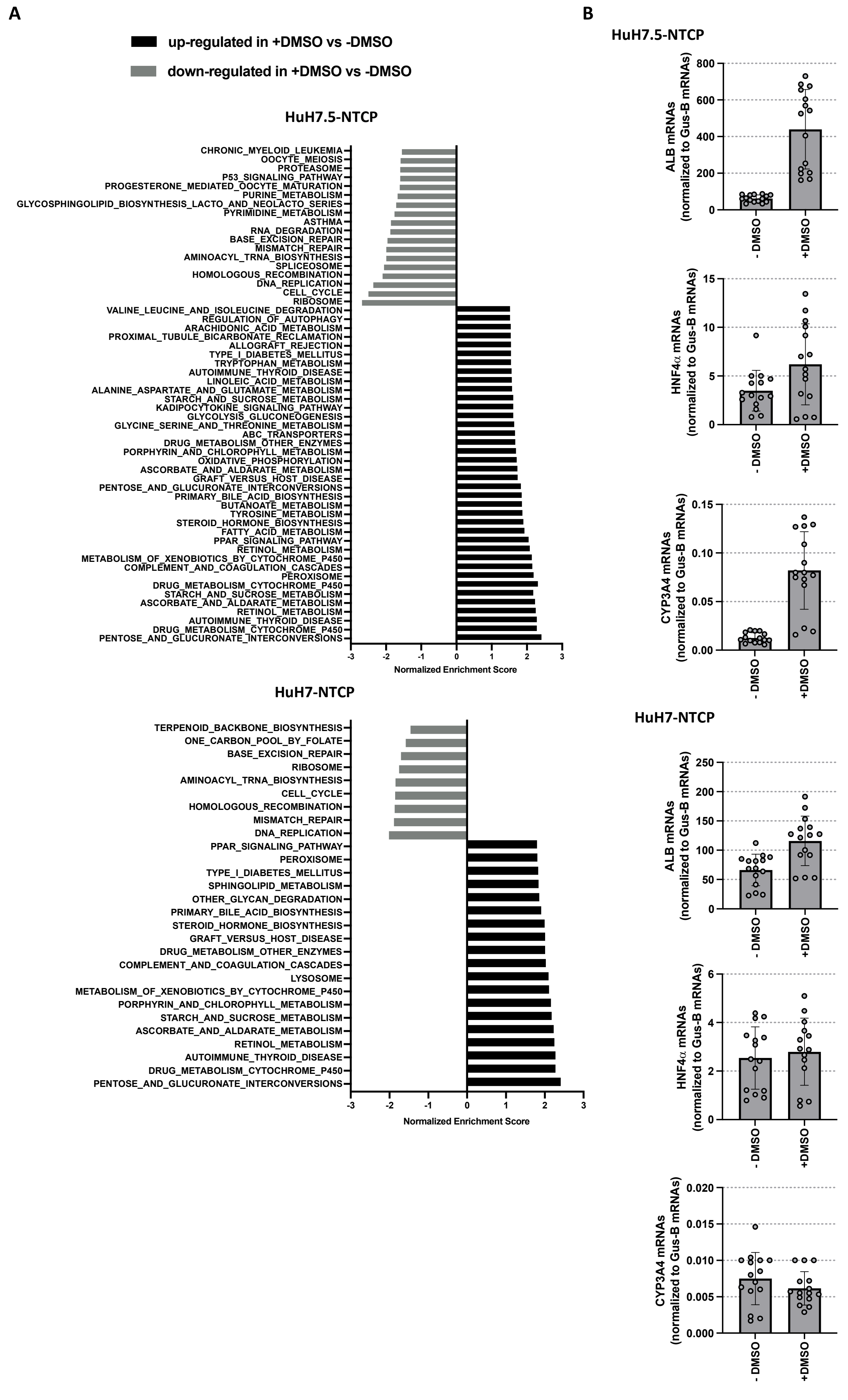

Fig. S1: DMSO treatment of HuH7.5-NTCP and HuH7-NTCP cells allows expression of liver-specific genes. (A) HuH7-NTCP or HuH7.5-NTCP cells have been seeded and treated or not with 2% DMSO as indicated in Figure 1A before analyses. Cells were lysed and total RNAs were analyzed by RNA sequencing. Pathway analyses were performed with KEGG and are presented for each cell line as normalized enrichment score. (B) Cells were lysed and total RNAs were analyzed by specific RT-qPCR analyses. Levels of target mRNAs were normalized to the levels of Gus-B mRNAs. Data are the mean +/-SD of 3 to 5 independent experiments each performed with 2 or 3 biological replicates. Dots represents the different biological replicates from all the experiments.

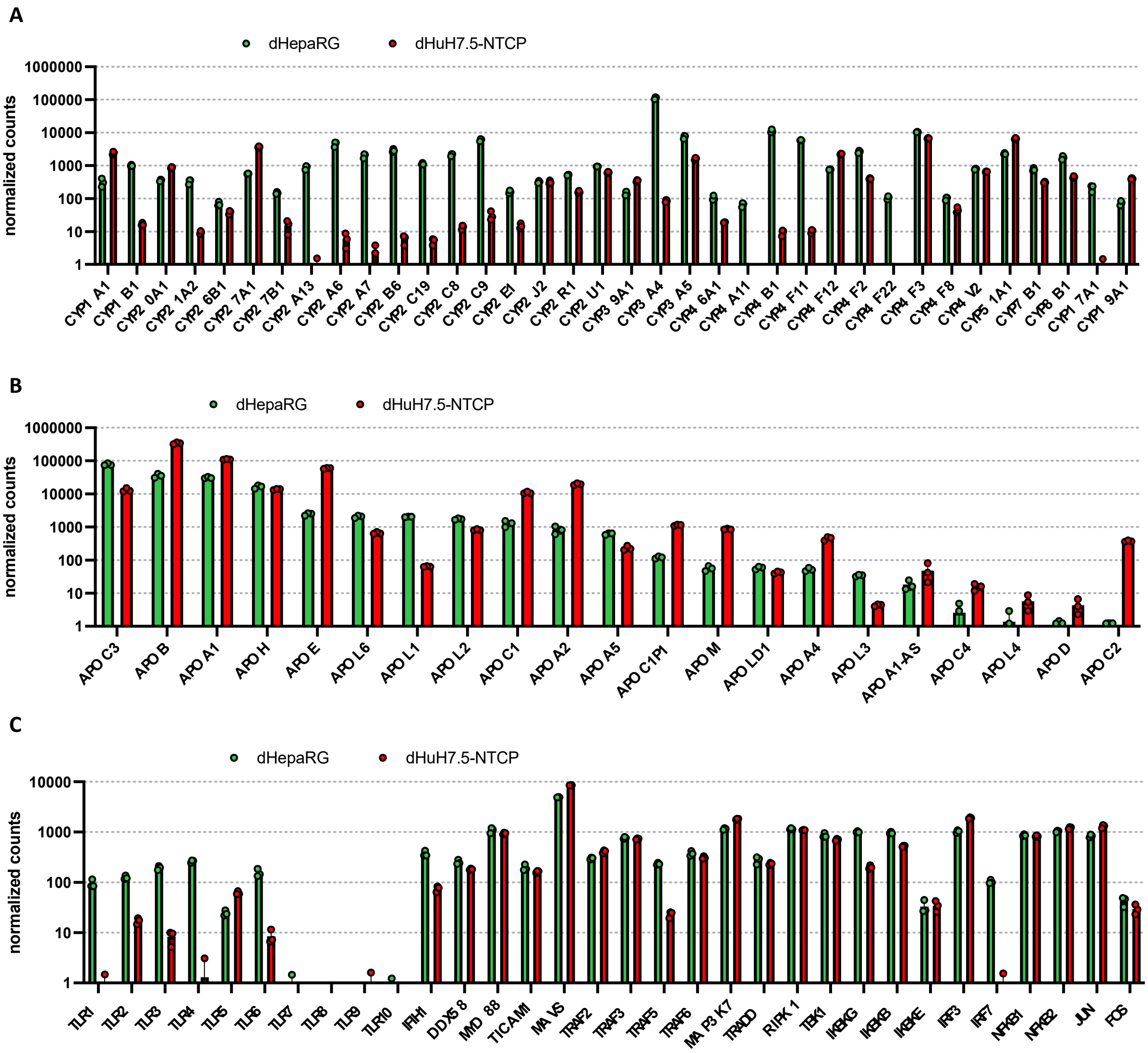

Fig. S2: Comparison of gene expression in dHepaRG and dHuH7.5-NTCP cells. Total RNAs extracted from dHepaRG or dHuH7.5-NTCP cells were analyzed by RNA sequencing. Normalized counts for (A) cytochrome related, (B) apolipoprotein-related or (C) RLR pathway related transcript are presented. Results are the mean +/- SD of 3 biological replicates. Dots represents the different biological replicates.

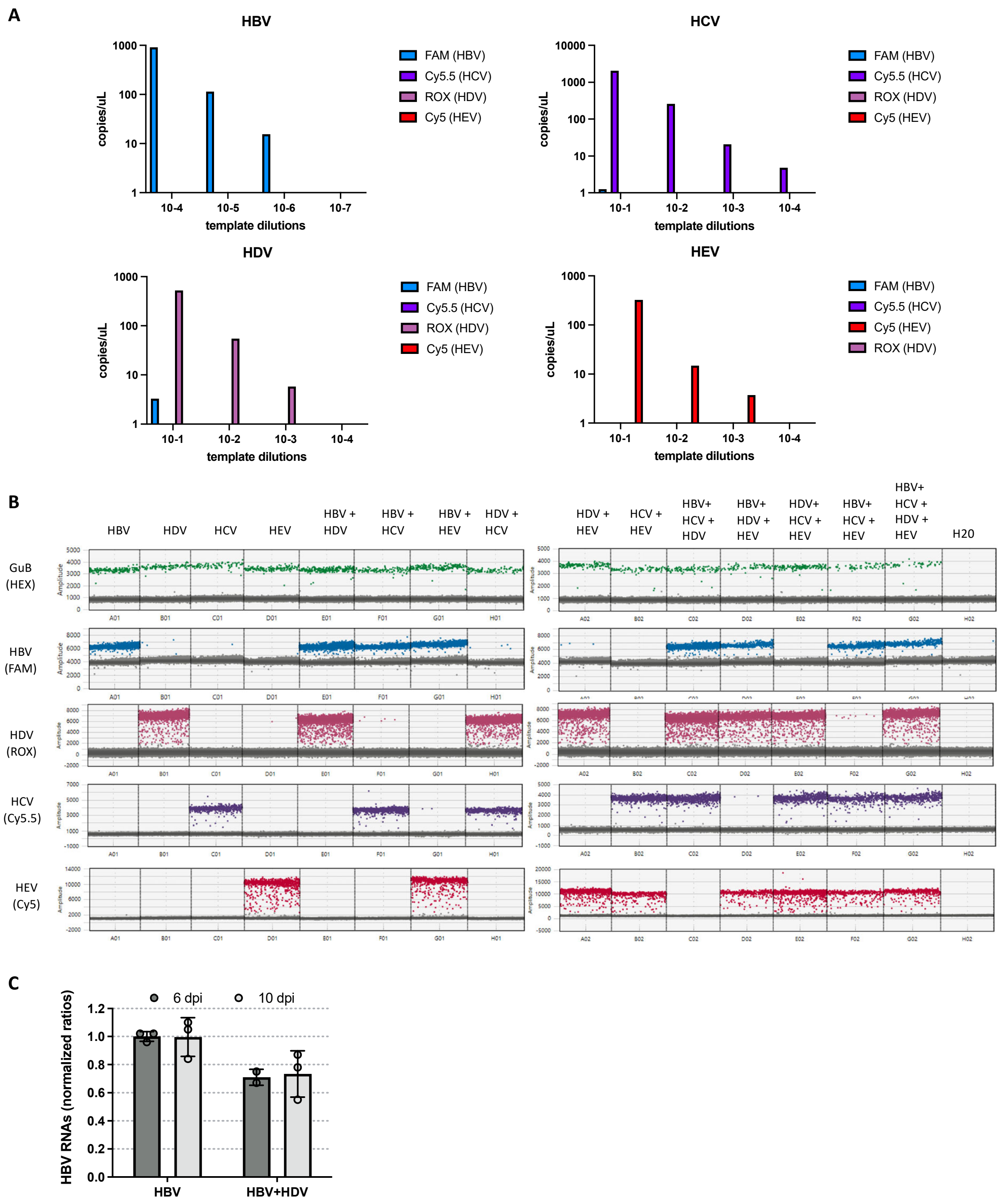

Fig. S3: Multiplex RT-ddPCR assay for simultaneous detection of HBV, HCV, HDV and HEV. (A) To determine the specificity of the assay, total nucleic acids from HBV, HCV, HEV and HDV inocula were extracted and RT-ddPCR analyses were performed with serial dilutions of each DNA/cDNA and a ddPCR reaction mix containing the 4 different sets of primers and probes to detect the 4 viral nucleic acids. (B) dHuH7.5-NTCP were inoculated with HCV, HDV, HEV, HBV or different combination of the 4 viruses at the same time. At days 10 post-inoculation, cells were lysed and the levels of viral RNAs were assessed by multiplex RT-ddPCR with specific probes. (C) dHuH7.5-NTCP were inoculated with HBV or HBV+HDV. At the indicated day post-inoculation, cells were lysed and the levels of HBV RNAs were assessed by multiplex RT-ddPCR with specific probes. Data (normalized to GusB mRNA and the HBV alone condition) are the mean +/-SD one two to three biological replicate.

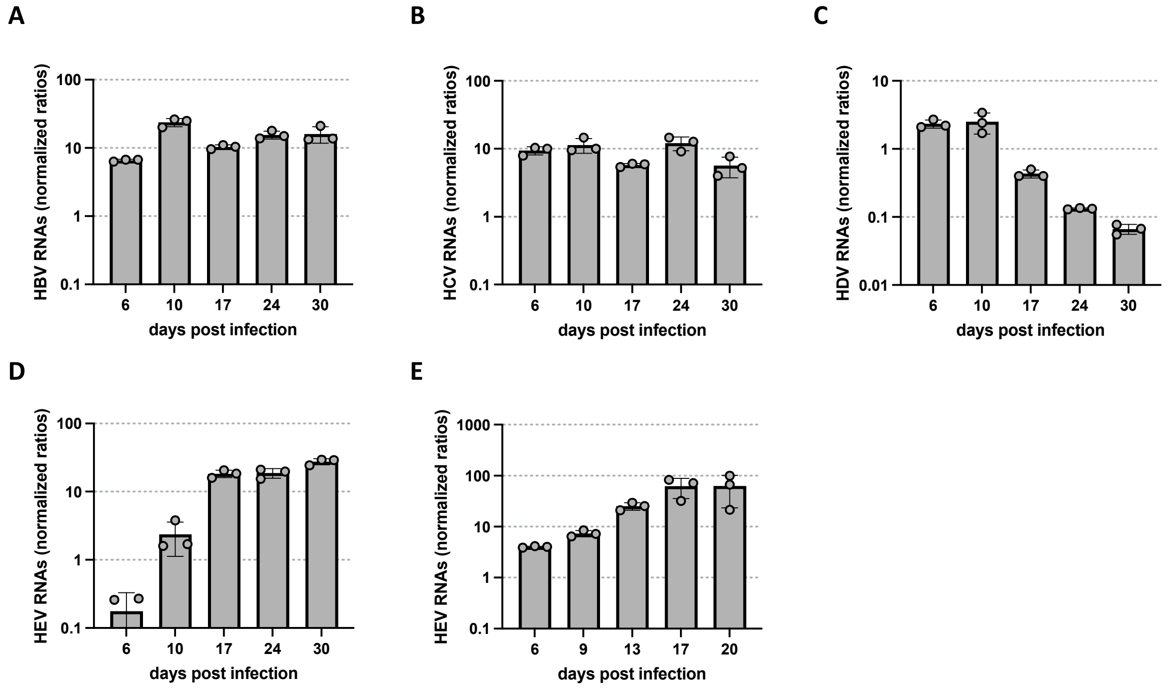

Fig. S4: Infections of dHuH7.5-NTCP cells by HBV, HCV, HDV and different HEV strains. HuH7.5-NTCP were differentiated with 2% DMSO for a week before inoculations with (A) HBV, (B) HCV, (C) HDV, (D) extracellular dHepaRG cells derived HEV-3 i.e. enveloped virus or (E) intracellular cell culture derived p6 HEV-3 i.e. naked virus. At the indicated days post-infection cells were lysed and the levels of intracellular viral RNAs were assessed by RT-qPCR. Data are the mean +/- SD of three biological replicate. Dots represents the different biological replicates.

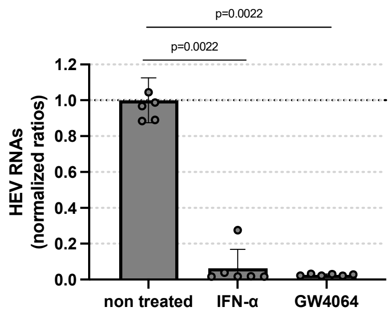

Fig. S5: Treatments with IFN-a or FXR-ligands abrogate HEV infection in dHuH7.5-NTCP cells. HuH7.5-NTCP were differentiated with 2% DMSO for a week before inoculations with intracellular cell culture derived p6 HEV-3 i.e. naked virus. Three days post-inoculation, cells were treated or not with IFN-α (500 IU/mL) or GW4064 (10 µM) for 10 days. Cells were lysed and the levels of intracellular HEV RNAs were assessed by RT-qPCR. Data are the mean +/- SD of two independent experiments each performed with three biological replicate. Dots represents the different biological replicates from all the experiments.

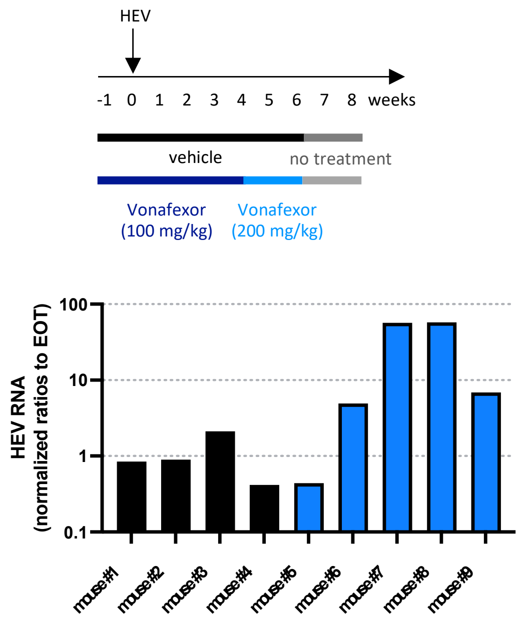

Fig. S6: Viral kinetics after cessation of Vonafexor treatment. HuHep mice were treated and infected with HEV-3 as indicated. Two weeks after arrest of treatment (8 weeks post-infection), levels of HEV RNAs in the stool of mice were quantified by qRT-PCR and normalized to levels at end of treatment (EOT).

Original Western blots

Figure 1C

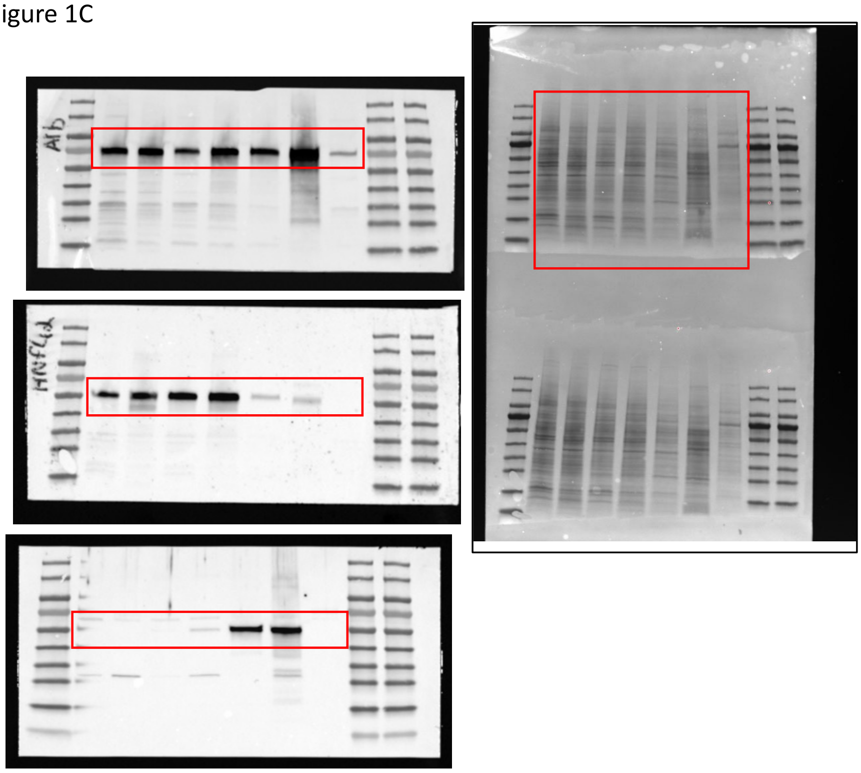

Figure 2B

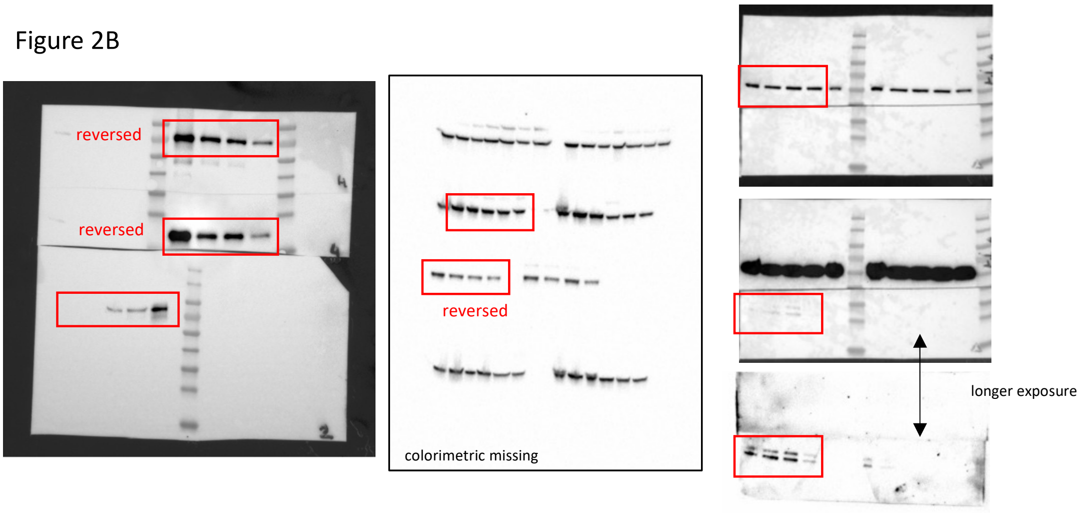

Supplementary tables

mRNAs	Forward primer sequence (5'-3')	Reverse primer sequence (3'-5')	Probe for ddPCR
Albumin	CTGCACAGAATCCTTGGTGAAC	TTTGGGAACGTATGTTTCATCG	
CYP3A4	CTTCATCCAATGGACTGCATAAAT	TCCCAAGTATAACACTCTACACAGACAA	
HNF4α	GAGTGGGCCAAGTACA	GGCTTTGAGGTAGGCATA	
RSAD2	CTTTGTGCTGCCCCTTGAG	TCCATACCAGCTTCCTTAAGCAA	
IL-6	TCGAGCCCACCGGGAACGAA	GCAACTGGACCGAAGGCGCT	
HBV	ACCGAATGTTGCCCAAGGTC	TATGCCTCAAGGTCGGTCGT	[FAM]-TCAACGACCGACCTTGAGGCA[BHQ1]
HDV	CGGGCCGGCTACTCTTCT	AAGGAAGGCCCTCGAGAACA	[ROX]-TGCCTCCCGCCGATAGCTGCT-[BHQ2]
HCV	CTCCCGGGGCACTCGCAAGC	GTCTAGCCATGGCGTTAGTA	[Cy5.5]-GCCTCCAGGCCCCCCCCTCC-[BHQ3]
HEV (quasi-enveloped)	GGTGGTTTCTGGGGTGAC	AGGGGTTGGTTGGATGAA	[Cy5]TGATTCTCAGCCCTTCGC-[BHQ2]
Gus B	CGTGGTTGGAGAGCTCATTTGGAA	ATTCCCCAGCACTCTCGTCGGT	[HEX]-CGTGTCCCTTCCTCCCCGAG-IB(R)FQ
HEV (naked)	ATTGGCCAGAAGTTGGTTTTCAC	CCGTGGCTATAATTGTGGTCT	

Table S1. Primers and probes for qPCR or ddPCR analyses.

Gene Name	Ensembl	Uniprot	Expressed in HuH7.5-NTCP cells	Expressed in HuH7-NTCP cells
A1BG	ENSG00000121410	P04217	X	X
A1CF	ENSG00000148584	Q9NQ94	X	X
ABCB11	ENSG00000073734	O95342	X	X
ABCB4	ENSG00000005471	P21439	X	X
ACADSB	ENSG00000196177	P45954	X	X
ACAT2	ENSG00000120437	Q9BWD1	X	X
ACOT12	ENSG00000172497	Q8WYK0	X	X
ACOX2	ENSG00000168306	Q99424	X	X
ACSM5	ENSG00000183549	Q6NUN0	X	X
ADH1A	ENSG00000187758	P07327	X	X
ADH4	ENSG00000198099	P08319	X	X
ADH6	ENSG00000172955	P28332	X	X
AFM	ENSG00000079557	P43652	X	X
AFP	ENSG00000081051	P02771	X	X
AGMO	ENSG00000187546	Q6ZNB7	X	X
AGT	ENSG00000135744	P01019	X	X
AGXT	ENSG00000172482	P21549	X	X
AHSG	ENSG00000145192	P02765	X	X
AKR1C4	ENSG00000198610	P17516	X	X
AKR1D1	ENSG00000122787	P51857	X	X
ALB	ENSG00000163631	P02768	X	X
AMBP	ENSG00000106927	P02760	X	X
ANG	ENSG00000214274	P03950	X	X
ANGPTL3	ENSG00000132855	Q9Y5C1	X	X
ANGPTL6	ENSG00000130812	Q8NI99	X	X
ANGPTL8	ENSG00000130173	Q6UXH0	X	X
APCS	ENSG00000132703	P02743	X	X

APOA1	ENSG00000118137	P02647	X	X
APOA2	ENSG00000158874	P02652	X	X
APOA5	ENSG00000110243	Q6Q788	X	X
APOC1	ENSG00000130208	P02654	X	X
APOC2	ENSG00000234906	P02655	X	X
APOC3	ENSG00000110245	P02656	X	X
APOC4	ENSG00000267467	P55056	X	X
APOC4-APOC2	ENSG00000224916		No	No
APOF	ENSG00000175336	Q13790	X	X
APOH	ENSG00000091583	P02749	X	X
APOM	ENSG00000204444	O95445	X	X
AQP9	ENSG00000103569	O43315	No	No
ARG1	ENSG00000118520	P05089	X	X
ARID3C	ENSG00000205143	A6NKF2	X	X
ARMC6	ENSG00000105676	Q6NXE6	X	X
ASGR1	ENSG00000141505	P07306	X	X
ASGR2	ENSG00000161944	P07307	X	X
ASL	ENSG00000126522	P04424	X	X
ATF5	ENSG00000169136	Q9Y2D1	X	X
BAAT	ENSG00000136881	Q14032	X	X
BCHE	ENSG00000114200	P06276	X	X
BDH1	ENSG00000161267	Q02338	X	X
C1S	ENSG00000182326	P09871	X	X
C2	ENSG00000166278	P06681	X	X
C3	ENSG00000125730	P01024	X	X
C4BPA	ENSG00000123838	P04003	X	X
C4BPB	ENSG00000123843	P20851	X	X
C5	ENSG00000106804	P01031	X	X
C6	ENSG00000039537	P13671	X	X
C8A	ENSG00000157131	P07357	X	X
C8B	ENSG00000021852	P07358	X	X
C8G	ENSG00000176919	P07360	X	X
C9	ENSG00000113600	P02748	X	X
CA5A	ENSG00000174990	P35218	X	X
CCDC152	ENSG00000198865	Q4G0S7	X	X
CCL16	ENSG00000275152	O15467	X	X
CES1	ENSG00000198848	P23141	X	X
CFB	ENSG00000243649	P00751	X	X
CFH	ENSG00000000971	P08603	X	X
CFHR1	ENSG00000244414	Q03591	X	X
CFHR2	ENSG00000080910	P36980	X	X
CFHR3	ENSG00000116785	Q02985	X	X
CFHR4	ENSG00000134365	Q92496	X	X
CFHR5	ENSG00000134389	Q9BXR6	X	X
CFI	ENSG00000205403	P05156	X	X
CLEC1B	ENSG00000165682	Q9P126	X	No
COLEC10	ENSG00000184374	Q9Y6Z7	X	X
CP	ENSG00000047457	P00450	X	X
CPB2	ENSG00000080618	Q96IY4	X	X
CPN1	ENSG00000120054	P15169	X	X
CPN2	ENSG00000178772	P22792	X	X

CPS1	ENSG00000021826	P31327	X	X
CRP	ENSG00000132693	P02741	X	X
CXCL2	ENSG00000081041	P19875	X	X
CYP1A2	ENSG00000140505	P05177	X	X
CYP26A1	ENSG00000095596	O43174	X	X
CYP2A13	ENSG00000197838	Q16696	X	X
CYP2A6	ENSG00000255974	P11509	X	X
CYP2A7	ENSG00000198077	P20853	X	X
CYP2B6	ENSG00000197408	P20813	X	X
CYP2C19	ENSG00000165841	P33261	X	X
CYP2C8	ENSG00000138115	P10632	X	X
CYP2C9	ENSG00000138109	P11712	X	X
CYP2D6	ENSG00000100197	P10635	X	X
CYP2E1	ENSG00000130649	P05181	X	X
CYP39A1	ENSG00000146233	Q9NYL5	X	X
CYP3A4	ENSG00000160868	P08684	X	X
CYP3A43	ENSG00000021461	Q9HB55	X	X
CYP3A7	ENSG00000160870	P24462	X	X
CYP3A7-CYP3A51P	ENSG00000282301		No	No
CYP4A22	ENSG00000162365	Q5TCH4	X	No
CYP7A1	ENSG00000167910	P22680	X	No
CYP8B1	ENSG00000180432	Q9UNU6	X	X
DCXR	ENSG00000169738	Q7Z4W1	X	X
DECR2	ENSG00000242612	Q9NUI1	X	X
DHODH	ENSG00000102967	Q02127	X	X
DNAJC22	ENSG00000178401	Q8N4W6	X	X
DNAJC25	ENSG00000059769	Q9H1X3	X	X
ENSG00000273047	ENSG00000273047		No	No
ENSG00000273171	ENSG00000273171		No	No
ENSG00000289697	ENSG00000289697		No	No
EPO	ENSG00000130427	P01588	X	X
EVA1A	ENSG00000115363	Q9H8M9	X	X
F11	ENSG00000088926	P03951	X	X
F12	ENSG00000131187	P00748	X	X
F13B	ENSG00000143278	P05160	X	X
F2	ENSG00000180210	P00734	X	X
F7	ENSG00000057593	P08709	X	X
F9	ENSG00000101981	P00740	X	X
FCN2	ENSG00000160339	Q15485	No	No
FETUB	ENSG00000090512	Q9UGM5	X	X
FGA	ENSG00000171560	P02671	X	X
FGB	ENSG00000171564	P02675	X	X
FGF21	ENSG00000105550	Q9NSA1	X	X
FGG	ENSG00000171557	P02679	X	X
FGL1	ENSG00000104760	Q08830	X	X
FMO3	ENSG00000007933	P31513	X	X
FMO5	ENSG00000131781	P49326	X	X
FTCD	ENSG00000160282	O95954	X	X
FUOM	ENSG00000148803	A2VDF0	X	X
GBP7	ENSG00000213512	Q8N8V2	X	X
GC	ENSG00000145321	P02774	X	X

GCHFR	ENSG00000137880	P30047	X	X
GCKR	ENSG00000084734		X	X
GDF2	ENSG00000263761	Q9UK05	No	No
GGCX	ENSG00000115486	P38435	X	X
GLDC	ENSG00000178445	P23378	X	X
GLS2	ENSG00000135423	Q9UI32	X	X
GLTPD2	ENSG00000182327	A6NH11	X	X
GLYCTK	ENSG00000168237	Q8IVS8	X	X
GOLT1A	ENSG00000174567	Q6ZVE7	X	X
GPLD1	ENSG00000112293	P80108	X	X
GSTZ1	ENSG00000100577	O43708	X	X
GYS2	ENSG00000111713	P54840	No	No
HAAO	ENSG00000162882	P46952	X	X
HABP2	ENSG00000148702	Q14520	X	X
HAMP	ENSG00000105697	P81172	X	No
HAO1	ENSG00000101323	Q9UJM8	X	X
HGFAC	ENSG00000109758	Q04756	X	X
HMGCS2	ENSG00000134240	P54868	X	X
HP	ENSG00000257017	P00738	X	X
HPD	ENSG00000158104	P32754	X	X
HPR	ENSG00000261701	P00739	X	X
HPX	ENSG00000110169	P02790	X	X
HRG	ENSG00000113905	P04196	X	X
HSD11B1	ENSG00000117594	P28845	No	No
HSD17B13	ENSG00000170509	Q7Z5P4	X	X
HSD17B6	ENSG00000025423	O14756	X	X
HSD3B7	ENSG00000099377	Q9H2F3	X	X
IGFALS	ENSG00000099769	P35858	X	X
IGFBP1	ENSG00000146678	P08833	X	X
IL1RAP	ENSG00000196083	Q9NPH3	X	X
IL27	ENSG00000197272	Q8NEV9	X	X
INHBC	ENSG00000175189	P55103	X	X
INHBE	ENSG00000139269	P58166	X	X
INSIG1	ENSG00000186480	O15503	X	X
ITIH1	ENSG00000055957	P19827	X	X
ITIH2	ENSG00000151655	P19823	X	X
ITIH3	ENSG00000162267	Q06033	X	X
ITIH4	ENSG00000055955	Q14624	X	X
KDM8	ENSG00000155666	Q8N371	X	X
KLKB1	ENSG00000164344	P03952	X	X
KNG1	ENSG00000113889	P01042	X	X
LBP	ENSG00000129988	P18428	X	X
LEAP2	ENSG00000164406	Q969E1	X	X
LECT2	ENSG00000145826	O14960	X	No
LEPR	ENSG00000116678	P48357	X	X
LIME1	ENSG00000203896	Q9H400	X	X
LIPC	ENSG00000166035	P11150	X	X
LPA	ENSG00000198670	P08519	X	X
LRG1	ENSG00000171236	P02750	X	X
MASP2	ENSG00000009724	O00187	X	X
MAT1A	ENSG00000151224	Q00266	X	X

MBL2	ENSG00000165471	P11226	X	X
MLXIPL	ENSG00000009950	Q9NP71	X	X
MST1	ENSG00000173531		X	X
MT1B	ENSG00000169688	P07438	X	X
MTHFD1	ENSG00000100714	P11586	X	X
MTHFS	ENSG00000136371	P49914	X	X
NADK2	ENSG00000152620	Q4G0N4	X	X
NEU4	ENSG00000204099	Q8WWR8	X	X
NR1I3	ENSG00000143257	Q14994	X	X
OAF	ENSG00000184232	Q86UD1	X	X
OIT3	ENSG00000138315	Q8WWZ8	X	X
ORM1	ENSG00000229314	P02763	X	X
ORM2	ENSG00000228278	P19652	X	X
OSGIN1	ENSG00000140961	Q9UJX0	X	X
OXER1	ENSG00000162881	Q8TDS5	X	X
PCSK9	ENSG00000169174	Q8NBP7	X	X
PECR	ENSG00000115425	Q9BY49	X	X
PGLYRP2	ENSG00000161031	Q96PD5	X	X
PLG	ENSG00000122194	P00747	X	X
PLGLB2	ENSG00000125551	Q02325	X	X
PNPLA3	ENSG00000100344	Q9NST1	X	X
PON1	ENSG00000005421	P27169	X	X
PON3	ENSG00000105852	Q15166	X	X
PRAMEF10	ENSG00000187545	O60809	No	No
PRAMEF33	ENSG00000237700	A0A0G2JMD5	No	No
PROC	ENSG00000115718	P04070	X	X
PROX1	ENSG00000117707	Q92786	X	X
PROZ	ENSG00000126231	P22891	X	X
PZP	ENSG00000126838	P20742	X	X
RBP4	ENSG00000138207	P02753	X	X
RDH16	ENSG00000139547	O75452	X	X
RNASE4	ENSG00000258818	P34096	X	X
RTP3	ENSG00000163825	Q9BQQ7	X	No
SAA1	ENSG00000173432	P0DJI8	X	X
SAA2	ENSG00000134339	P0DJI9	X	X
SAA2-SAA4	ENSG00000255071		No	No
SAA4	ENSG00000148965	P35542	X	X
SDS	ENSG00000135094	P20132	X	X
SERPINA1	ENSG00000197249	P01009	X	X
SERPINA10	ENSG00000140093	Q9UK55	X	X
SERPINA11	ENSG00000186910	Q86U17	X	X
SERPINA4	ENSG00000100665	P29622	X	X
SERPINA6	ENSG00000170099	P08185	X	X
SERPINA7	ENSG00000123561	P05543	X	X
SERPINC1	ENSG00000117601	P01008	X	X
SERPIND1	ENSG00000099937	P05546	X	X
SERPINF2	ENSG00000167711	P08697	X	X
SHBG	ENSG00000129214	P04278	X	X
SLC10A1	ENSG00000100652	Q14973	X	X
SLC13A5	ENSG00000141485	Q86YT5	X	X
SLC17A2	ENSG00000112337	O00624	X	X

SLC22A1	ENSG00000175003	O15245	X	X
SLC22A10	ENSG00000184999	Q63ZE4	X	X
SLC22A25	ENSG00000196600	Q6T423	X	X
SLC22A7	ENSG00000137204	Q9Y694	X	X
SLC22A9	ENSG00000149742	Q8IVM8	X	X
SLC25A13	ENSG00000004864	Q9UJS0	X	X
SLC25A47	ENSG00000140107	Q6Q0C1	X	X
SLC27A5	ENSG00000083807	Q9Y2P5	X	X
SLC2A2	ENSG00000163581	P11168	X	X
SLC38A4	ENSG00000139209	Q969I6	X	X
SLCO1B1	ENSG00000134538	Q9Y6L6	X	X
SLCO1B3	ENSG00000111700	Q9NPD5	X	X
SMLR1	ENSG00000256162	H3BR10	X	X
SPP2	ENSG00000072080	Q13103	X	X
SULT2A1	ENSG00000105398	Q06520	X	X
TAT	ENSG00000198650	P17735	X	X
TDO2	ENSG00000151790	P48775	X	X
TFR2	ENSG00000106327	Q9UP52	X	X
THPO	ENSG00000090534	P40225	X	X
TLCD4	ENSG00000152078	Q96MV1	X	X
TMEM176B	ENSG00000106565	Q3YBM2	X	X
TMPRSS6	ENSG00000187045	Q8IU80	X	X
TTC36	ENSG00000172425	A6NLP5	X	X
TTPA	ENSG00000137561	P49638	X	X
UGT1A3	ENSG00000288702	P35503	X	X
UGT1A4	ENSG00000244474	P22310	X	X
UGT2B10	ENSG00000109181	P36537	X	X
UGT2B4	ENSG00000156096	P06133	X	X
UPB1	ENSG00000100024	Q9UBR1	X	X
UROC1	ENSG00000159650	Q96N76	X	No
VTN	ENSG00000109072	P04004	X	X
ZGPAT	ENSG00000197114	Q8N5A5	X	X

Table S2: Expression of 263 liver-specific, protein-coding genes according to the Human Protein Atlas and RNA-seq data (GSE288204) *in HuH7-NTCP vs HuH7.5-NTCP cells*

Supplementary references

Author names in bold designate shared co-first authorship

[1] Alfaiate D, Lucifora J, Abeywickrama-Samarakoon N et al. HDV RNA replication is associated with HBV repression and interferon-stimulated genes induction in super-infected hepatocytes. Antiviral Res 2016;136:19-31.
[2] Gripon P, Rumin S, Urban S et al. Infection of a human hepatoma cell line by hepatitis B virus. Proc Natl Acad Sci U S A 2002;99:15655-15660.
[3] Lecluyse EL, Alexandre E. Isolation and culture of primary hepatocytes from resected human liver tissue. Methods Mol Biol 2010;640:57-82.
[4] Ladner SK, Otto MJ, Barker CS et al. Inducible expression of human hepatitis B virus (HBV) in stably transfected hepatoblastoma cells: a novel system for screening potential inhibitors of HBV replication. Antimicrob Agents Chemother 1997;41:1715-1720.
[5] **Bach C, Lucifora J**, Delphin M et al. A stable hepatitis D virus-producing cell line for host target and drug discovery. Antiviral Res 2023;209:105477.
[6] Delgrange D, Pillez A, Castelain S et al. Robust production of infectious viral particles in Huh-7 cells by introducing mutations in hepatitis C virus structural proteins. J Gen Virol 2007;88:2495-2503.
[7] Pellerin M, Hirchaud E, Blanchard Y et al. Characterization of a Cell Culture System of Persistent Hepatitis E Virus Infection in the Human HepaRG Hepatic Cell Line. Viruses 2021;13.
[8] **Todt D, Friesland M**, Moeller N et al. Robust hepatitis E virus infection and transcriptional response in human hepatocytes. Proc Natl Acad Sci U S A 2020;117:1731-1741.
[9] Nielsen SU, Bassendine MF, Martin C et al. Characterization of hepatitis C RNA-containing particles from human liver by density and size. J Gen Virol 2008;89:2507-2517.
[10] Sayed IM, Verhoye L, Cocquerel L et al. Study of hepatitis E virus infection of genotype 1 and 3 in mice with humanised liver. Gut 2017;66:920-929.
[11] Peck D, Crawford ED, Ross KN et al. A method for high-throughput gene expression signature analysis. Genome Biol 2006;7:R61.
[12] Uhlen M, Fagerberg L, Hallstrom BM et al. Proteomics. Tissue-based map of the human proteome. Science 2015;347:1260419.
[13] Taverniti V, Meiss-Heydmann L, Gadenne C et al. CAM-A-dependent HBV core aggregation induces apoptosis through ANXA1. JHEP Rep 2024;6:101134.
