# Supplementary material for: A novel in vitro system for simultaneous infections with hepatitis B, C, D and E viruses
Source: JHEP Rep. 2025 Feb 28;7(5):101383. doi: 10.1016/j.jhepr.2025.101383 (PMC11999259; doi:10.1016/j.jhepr.2025.101383)
Supplement: Multimedia component 2 [file mmc2.docx]

JHEP Reports CTAT methods

Tables for a “Complete, Transparent, Accurate and Timely account” (CTAT) are now mandatory for all revised submissions. The aim is to enhance the reproducibility of methods.

- Only include the parts relevant to your study
- Refer to the CTAT in the main text as ‘Supplementary CTAT Table’
- Do not add subheadings
- Add as many rows as needed to include all information
- Only include one item per row

If the CTAT form is not relevant to your study, please outline the reasons why:

- 1. **Antibodies**

| **Name** | **Citation** | **Supplier** | **Cat no.** | **Clone no.** |
| --- | --- | --- | --- | --- |
| Anti-γ-tubulin | / | Sigma | T6557 | GTU-88 |
| Anti-albumin | / | Dako | NA | - |
| Anti-ORF2 | / | Milipore | MAB8002 | 1E6 |
| Anti-HCV NS3 | / | Virogen | 217-A | 9-G2 |
| Anti-HCV Core | / | Thermofisher | MA1-7366 | D95B |
| Anti-HDAg | / | Eurogenetec | Home-made | NA |
| Anti-ApoB | / | Biodesign | H45640M | - |
| Anti-ApoB -HRP | / | Biodesign | K34005G | - |
| Polyclonal Goat anti-Rabbit immunoglobulins - HRP | / | DAKO | P044801-2 | - |
| Polyclonal Goat anti-Mouse immunoglobulins - HRP | / | DAKO | [P044701-2](https://www.agilent.com/store/productDetail.jsp?catalogId=P044701-2) | - |

- 1. **Cell lines**

| **Name** | **Citation** | **Supplier** | **Cat no.** | **Passage no.** | **Authentication test method** |
| --- | --- | --- | --- | --- | --- |
| HepaRG | PMID: 12432097 | Isolated in the lab of C. Trepo/F. Zoulim | NA | Below 20 | None |
| HuH7 | PMID: **11507197** | Christophe Seeger Fox Chase Center Philadelphia, USA | NA | Below 20 | None |
| HuH7.5 | Provided by Dr CM. Rice and described in PMID: **12438626** | Dr CM Rice, Rockfeller University | NA | Below 20 | None |
| HEK293 | NA | ThermoFisher scientific | R70007 | Below 20 | None |

- 1. **Organisms**

| **Name** | **Citation** | **Supplier** | **Strain** | **Sex** | **Age** | **Overall n number** |
| --- | --- | --- | --- | --- | --- | --- |
| uPA^+/+^-SCID mice | PMID: 15791625 | In house breeding | Alb-uPA^+/+^/CB-17/Icr-Prkdc^scid/scid^/Rj | Male/Female | 2 weeks | 9 |

- 1. **Sequence based reagents**

| **Name** | **Sequence** | **Supplier** |
| --- | --- | --- |
| Albumin | FW : CTGCACAGAATCCTTGGTGAAC  RV : TTTGGGAACGTATGTTTCATCG | Eurogentec |
| CYP3A4 | FW :CTTCATCCAATGGACTGCATAAAT  RV : TCCCAAGTATAACACTCTACACAGACAA | Eurogentec |
| HNF4a | FW : GAGTGGGCCAAGTACA  RV : GGCTTTGAGGTAGGCATA | Eurogentec |
| RSAD2 | FW : CTTTGTGCTGCCCCTTGAG  RV : TCCATACCAGCTTCCTTAAGCAA | Eurogentec |
| IL6 | FW : TCGAGCCCACCGGGAACGAA  RV : GCAACTGGACCGAAGGCGCT | Eurogentec |
| HBV | FW : ACCGAATGTTGCCCAAGGTC  RV : TATGCCTCAAGGTCGGTCGT  Probe : [FAM]-TCAACGACCGACCTTGAGGCA-[BHQ1] | Eurogentec |
| HDV | FW : CGGGCCGGCTACTCTTCT  RV : AAGGAAGGCCCTCGAGAACA  Probe : [ROX]-TGCCTCCCGCCGATAGCTGCT- [BHQ2] | Eurogentec |
| HCV | FW : CTCCCGGGGCACTCGCAAGC  RV : GTCTAGCCATGGCGTTAGTA  Probe : [Cy5.5]-GCCTCCAGGCCCCCCCCTCC-[BHQ3] | Eurogentec |
| HEV (quasi-enveloped) | FW : GGTGGTTTCTGGGGTGAC  RV : AGGGGTTGGTTGGATGAA  Probe : [Cy5]-TGATTCTCAGCCCTTCGC-[BHQ2] | Eurogentec |
| HEV (naked) | FW : ATTGGCCAGAAGTTGGTTTTCAC  RV : CCGTGGCTATAATTGTGGTCT | Eurogentec |
| Gus B | FW : CGTGGTTGGAGAGCTCATTTGGAA  RV : ATTCCCCAGCACTCTCGTCGGT  Probe : [HEX]-CGTGTCCCTTCCTCCCCGAG-IB(R)FQ | Eurogentec |

- 1. **Biological samples**

| **Description** | **Source** | **Identifier** |
| --- | --- | --- |
| Primary Human Hepatocytes isolated from liver resections | Centre Léon Bérard, centre hospitalier Lyon-Sud | AC 2013-1871, DC 2013 – 1870, AFNOR NF 96 900 sept 2011 |

- 1. **Deposited data**

| **Name of repository** | **Identifier** | **Link** |
| --- | --- | --- |
| Gene Expression Omnibus | GSE288204 | <https://www.ncbi.nlm.nih.gov/geo/query/acc.cgi?acc=GSE288204> |
| Gene Expression Omnibus | GSE288203 | <https://www.ncbi.nlm.nih.gov/geo/query/acc.cgi?acc=GSE288203> |

- 1. **Software**

| **Software name** | **Manufacturer** | **Version** |
| --- | --- | --- |
| GraphPad Prism | GraphPad Software | 10 |
| Image Lab | Bio-Rad | 6.1 |
| QuantStudio TM Real time PCR software | Applied Biosystem | 2.6.0 |
| QX Manager | Bio-Rad | 2.1 |

- 1. **Other (*e.g*. drugs, proteins, vectors etc.)**

| Pam3CSK4 | Invivogen | Cat n#tlrl pms |
| --- | --- | --- |
| Riboxxol | Ribox | Cat n#A-00102 |
| LPS | Invivogen | Cat n#tlrl smlps |
| Poly IC HMW | Invivogen | Cat n# tlrl pic5 |
| IFN-a | Roche | Roferon |
| Sofosbuvir | Selleckchem | Cat n#S2794 |
| GW4064 | Selleckchem | Cat n#S2782 |
| Vonafexor | Enyo Pharma | Special synthesis |

- 1. **Please provide the details of the corresponding methods author for the manuscript:**

- Dr. Julie Lucifora ([julie.lucifora@inserm.fr](mailto:julie.lucifora@inserm.fr)), INSERM, 21 Avenue Tony Garnier 69007 Lyon, France, 0033437282401

2.0 Please confirm for randomised controlled trials all versions of the clinical protocol are included in the submission. These will be published online as supplementary information.
