# Supplementary material for: A novel in vitro system for simultaneous infections with hepatitis B, C, D and E viruses
Source: JHEP Rep. 2025 Feb 28;7(5):101383. doi: 10.1016/j.jhepr.2025.101383 (PMC11999259; doi:10.1016/j.jhepr.2025.101383)
Supplement: Multimedia component 4 [file mmc4.pdf]

A novel *in vitro* system for simultaneous infections with hepatitis B, C, D and E viruses

Authors

Roxanne Fouillé, Eloi R. Verrier, Amse De Meyer, …, Philip Meuleman, David Durantel, Julie Lucifora

Correspondence

julie.lucifora@inserm.fr (J. Lucifora), david.durantel@inserm.fr (D. Durantel).

Graphical abstract

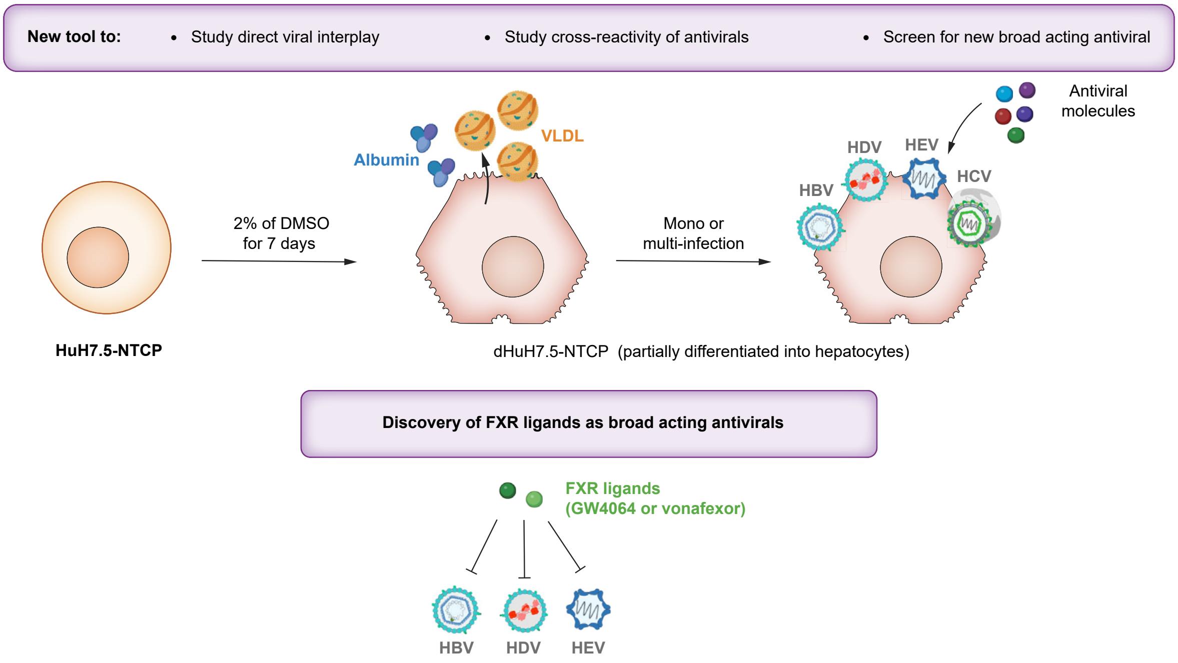

Highlights:

- HuH7.5-NTCP cells can be partially differentiated into hepatocyte-like cells using DMSO.
- dHuH7.5-NTCP allow replication of HBV, HDV, HCV and HEV for at least 4 weeks after mono or multiple infections.
- Treatment with FXR agonists leads to a reduction of HBV, HDV and HEV infection.

Impact and implications:

Hepatitis virus infections caused by HBV, HCV, HDV, and HEV represent a global health threat. Treatment options remain limited, notably due to the lack of knowledge about molecular virus-host interactions. Moreover, the interplay between these four viruses in the context of co-infections remains unknown. In this study, we report the first *in vitro* system that allows for mono and multi-infections with these four viruses and characterize the broad antiviral activity of farnesoid X receptor agonists, paving the way for the development of new strategies for viral cure.

https://doi.org/10.1016/j.jhepr.2025.101383
© 2025 The Author(s). Published by Elsevier B.V. on behalf of European Association for the Study of the Liver (EASL). This is an open access article under the CC BY license (http://creativecommons.org/licenses/by/4.0/).

JHEP|Reports

A novel *in vitro* system for simultaneous infections with hepatitis B, C, D and E viruses

Roxanne Fouillé[1], Eloi R. Verrier[2], Amse De Meyer[3], Lieven Verhoye[3], Maud Michelet[4], Romain Barnault[1], Caroline Pons[1], Olivier Diaz[1], Michel Rivoire[5], Guillaume Passot[6], Eike Steinmann[7], Heiner Wedemeyer[8,11], Anna Salvetti[1], Nicole Pavio[9], Virginie Doceul[9], Raphaël Darteil[10], Philip Meuleman[3], David Durantel[1,*,‡], Julie Lucifora[1,*,‡]

JHEP Reports **2025**. vol. 7 | 1–7

Check for updates

Background & Aims: The liver, and more precisely hepatocytes, can be infected by several hepatotropic viruses, including HBV, HDV, HCV and HEV, with chronic infection leading to end-stage liver diseases. Since no *in vitro* model allowing multi-infections with the four viruses is reported, limited data are available on their interplay as well as on the potential cross-reactivity of antivirals in multi-infection cases. The aim of our study was to set up such a model.

Methods: HuH7.5-NTCP cells were cultured with 2% DMSO (dimethyl sulfoxide) for 1 week to allow partial differentiation into hepatocytes (dHuH7.5-NTCP) before infection with the different viruses and treatment with known antiviral molecules.

Results: We observed increased expression of liver specific transcripts and production of ApoB containing VLDL in dHuH7.5-NTCP cells and replication of HBV, HDV, HCV and HEV for at least 4 weeks after mono or multiple infections. We recapitulated the known antiviral effect of sofosbuvir on HCV and HEV (>90% reduction in the levels of intracellular viral RNAs, p <0.0005) and of IFN-α on HCV, HEV and HDV (80% reduction in the levels of intracellular viral RNAs, p <0.0005). Besides its already described antiviral effect on HBV and HDV, we observed that GW4064, a farnesoid X receptor (FXR) agonist, also strongly inhibited HEV replication (85 to 95% reduction in the levels of intracellular HEV RNAs, p <0.0005). Using HEV-infected HuHep mice, we confirmed the antiviral effect of vonafexor, an FXR agonist, that is currently being tested clinically against HBV/HDV.

Conclusions: We set-up the first *in vitro* model allowing multi-infections with hepatitis viruses that can be used for broad drug screening and highlighted FXR ligands as potential broad-acting antivirals.

© 2025 The Author(s). Published by Elsevier B.V. on behalf of European Association for the Study of the Liver (EASL). This is an open access article under the CC BY license (http://creativecommons.org/licenses/by/4.0/).

Introduction

The liver, and in particular hepatocytes, are the target of several pathogens, including viruses such as HBV, HCV, HDV and HEV, which may impair their essential functions and trigger liver inflammation, fibrosis, cirrhosis, hepatocellular carcinoma and eventually lead to liver failure.[1] If HCV infection can be cured with specific direct-acting antivirals (DAAs), chronic HBV and HDV infections can only be contained using either nucleos(t)ide analogues or bulevirtide, but without viral clearance.[1] Ribavirin or, in some specific cases, pegylated–IFN–α, are used in clinic against chronic hepatitis E, but failures are often reported.[1,2] Very few epidemiological studies are available concerning multi-infections with several hepatitis viruses. Apart from the well-known HBV/HDV co-infections, infections with double or triple viruses have been reported[3–6] and these cases, as well as quadruple infections, are probably underestimated due to deficient diagnosis, in particular for HEV and HDV. Importantly, multi-infections with hepatitis viruses increase the risk of fulminant hepatitis,[7] and severe liver diseases.[8,9] Although HBV, HCV, HDV and HEV infections are restricted to hepatocytes, very few data are available concerning their potential interplay. Beside the well documented interference of HDV on HBV,[10,11] conflicting data have been reported on the interactions between other hepatitis viruses. It has been suggested that HCV may dominate HBV, HDV and HEV *in vivo*,[12,13] but HBV and HCV were found to replicate in the same cell without interference in HuH7 cells[14] and a case report described HCV clearance during acute HBV/HDV super-infection.[15] HEV was once found associated with repressed HBV replication,[16] whereas a case report described an asymptomatic HEV superinfection followed by a flare in HBV replication in an HBsAg carrier without signs of HBV replication for 8 years.[17] It is therefore not clear if and how one hepatitis virus would negatively interfere with or boost the replication of other hepatitis viruses in case of multi-infections *in vivo*. Moreover, the effects of the current antiviral treatments on co- or multi-infections are unknown and concerns about viral reactivation after clearance of one infection among the others have been raised. Finally, new antiviral strategies against HBV, HDV and HEV are needed, including broadly acting therapies that would ease the management of multi-infected patients. In this

* Corresponding authors. Address: CIRI – 21 avenue Tony Garnier, 69007 Lyon, France.
E-mail addresses: julie.lucifora@inserm.fr (J. Lucifora), david.durantel@inserm.fr (D. Durantel).
‡ Both contributed equally
https://doi.org/10.1016/j.jhepr.2025.101383

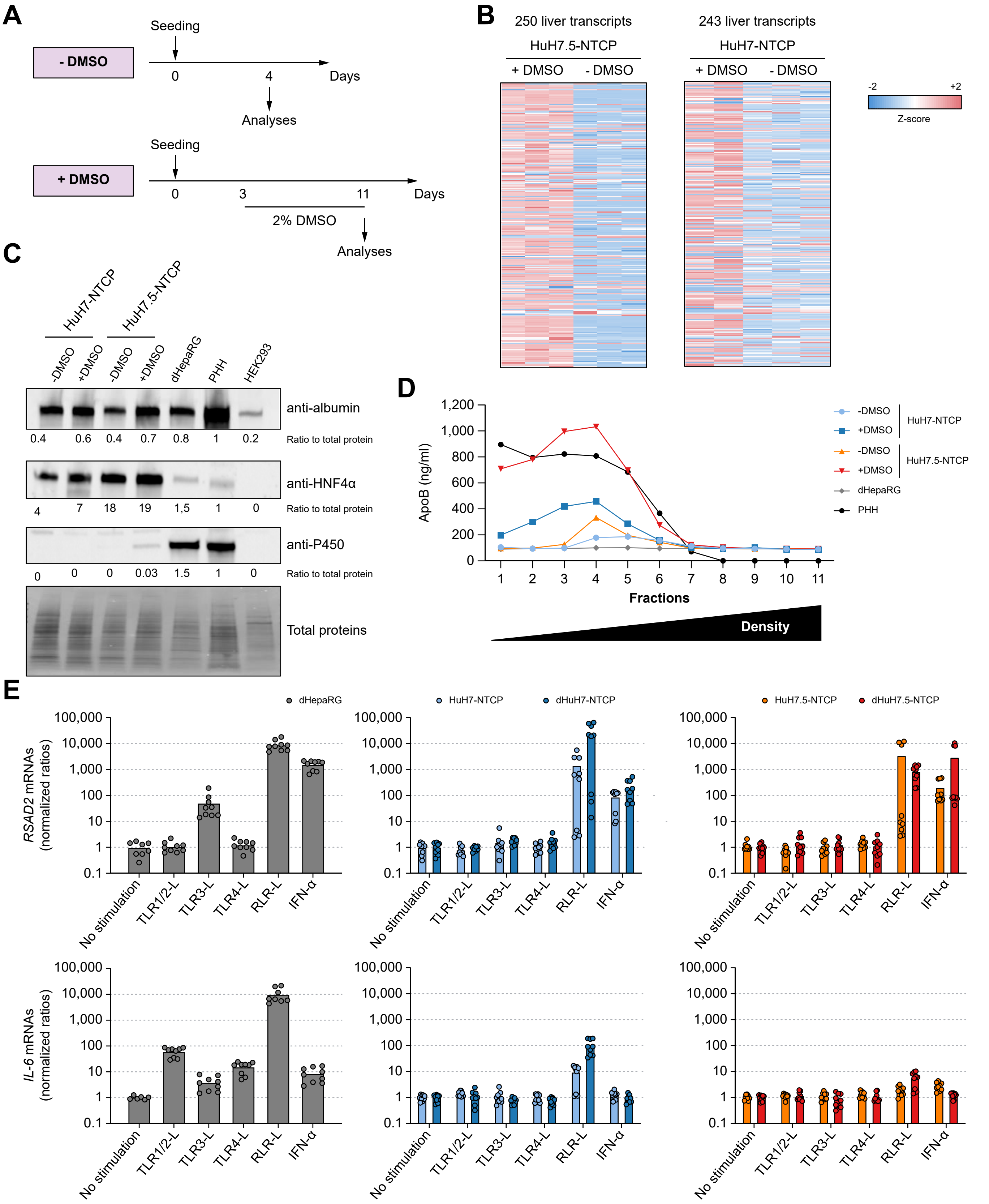

Fig. 1. DMSO treatment of HuH7.5-NTCP cells enables their partial differentiation into hepatocyte-like cells. (A-E) HuH7-NTCP or HuH7.5-NTCP cells were seeded and treated or not with 2% DMSO. (B) Cells were lysed, total RNAs were extracted and gene expression was assessed by RNA sequencing (GSE288204). Relative liver-specific gene expression (Z-score) of up to 250 genes (see Table S1 for details) is presented. (C, D, E) HepaRG cells were differentiated for 4 weeks (dHepaRG) and PHHs seeded for 24 h before analyses. (C) Cells were lysed and levels of the indicated proteins were assessed by western blot. Results from a

context, it is urgent to develop a system allowing researchers to determine how antiviral drugs may influence hepatitis viruses that are not initially targeted by the treatment in case of multiple infections.

HBV, HCV, HDV and HEV naturally infect and replicate into highly differentiated, non-dividing, human hepatocytes. Therefore, we aimed to establish a relevant and scalable *in vitro* hepatocyte culture model supporting the replication of these four viruses. Although primary human hepatocytes (PHHs) are the gold standard for HBV and HDV *in vitro* studies,[18] few data have been reported on their susceptibility to HEV infection,[19] and permissiveness to HCV is contested. Moreover, the use of PHHs is problematic, due to inter-individual variability and difficult access. Similarly, differentiated HepaRG (dHepaRG) cells are susceptible to HBV, HDV, and HEV infections[20,21] but not to HCV infection. HuH7 cells and its derivate clone, HuH7.5, are susceptible to HCV and HEV[22,23] but do not express the HBV/HDV receptor NTCP.[24] Interestingly, HuH7 cells treated with dimethyl sulfoxide (DMSO) display a more differentiated phenotype.[25]

We therefore investigated whether a 1-week treatment of HuH7-NTCP and HuH7.5-NTCP cell cultures with 2% DMSO may lead to their differentiation into hepatocyte like-cells and support the infection by these four major hepatitis viruses.

Materials and methods

The materials and methods used are described in the supplementary information.

Results

Through transcriptomic analyses in both cell lines, we identified DMSO-induced upregulated genes, mainly belonging to liver-specific pathways such as drug metabolism or primary bile acid biosynthesis (Fig. S1A). Analyses of relative liver gene expression according to the Human Protein Atlas showed that DMSO-treated HuH7-NTCP (dHuH7-NTCP) and HuH7.5-NTCP (dHuH7.5-NTCP) cells expressed most of the liver-specific genes (Fig. 1A,B, Table S2). In contrast, genes related to cell cycle or DNA replication pathways were downregulated upon treatment of cells with DMSO (Fig. S1A). Western blot and quantitative reverse-transcription PCR analyses indicated that, like PHHs and dHepaRG cells, dHuH7.5-NTCP cells expressed albumin, a blood protein secreted by differentiated hepatocytes, and HNF4α, an hepatocyte-specific transcription factor, essential for HBV RNA synthesis,[26] indicating the differentiation of cells. However, dHuH7.5-NTCP cells produced lower levels of CYP34A/P450 proteins than PHHs or dHepaRG cells (Fig. 1C and S1B). Additional transcriptomic analyses confirmed lower levels of several cytochrome transcripts in dHuH7.5-NTCP cells compared to dHepaRG cells (Fig. S2A). These data suggest that dHuH7.5-NTCP cells are less efficient in drug detoxification than PHHs and dHepaRG cells, a property that may help to unravel potential toxicity issues of antiviral molecules in drug screening assays. One of the key functions of hepatocytes is to release very low-density lipoprotein (VLDL) containing non-exchangeable ApoB into the bloodstream to supply body tissues with triglycerides. We observed that both dHuH7-NTCP and dHuH7.5-NTCP cells produced higher amounts of triglycerides in comparison to non-differentiated cells (Fig. 1D). Despite identical ApoB secretion before differentiation of these two cell lines, only dHuH7.5-NTCP cells were able to produce comparable levels of ApoB-containing VLDL to PHHs (Fig. 1D), thus suggesting an advantage of this subclone over the parental HuH7 cell line. Of note dHepaRG cells did not produce a measurable amount of ApoB-containing VLDL (Fig. 1D) and HuH7.5-NTCP cells displayed higher levels of most apolipoprotein transcripts compared to dHepaRG cells (Fig. S2B). An innate immune response to pathogens and especially pattern recognition receptor activation is also a strong feature of differentiated hepatocytes[27] and numerous immune pathways are impaired in hepatoma cell lines.[28] We therefore stimulated HuH7-NTCP and HuH7.5-NTCP cells with several pattern recognition receptor ligands to determine if they can recover some innate functions upon DMSO treatment (Fig. 1E). We confirmed the increased levels of *RSAD2* and *IL-6* transcripts upon TLR1/2, TLR3, TLR4, RLR ligands and IFN-α stimulations of dHepaRG cells.[28] In contrast, even after treatment with DMSO of HuH7-NTCP and HuH7.5-NTCP cells, levels of *RSAD2* transcripts were only increased following stimulations with RLR ligand and IFN-α, but not following TLR1/2, TLR3 or TLR4 stimulations. These data are in accordance with the levels of *TLR1, TLR2, TLR3* and *TLR4* transcripts being at least 1 log lower at steady state in dHuH7.5-NTCP cells compared to dHepaRG cells (Fig. S2C). Of note, compared to dHepaRG cells, the induction of *IL-6* transcripts was modest in HuH7-NTCP cells stimulated with an RLR ligand and very weak in HuH7.5-NTCP cells, irrespective of their differentiation status (Fig. 1E). The default of IL-6 production by dHuH7.5-NTCP cells might be due to lower levels of the *TRAF5* transcripts, required for NF-kB activation,[29] in dHuH7.5-NTCP cells compared to dHepaRG cells at steady state (Fig. S2C). Altogether, our data indicate that treatment with DMSO allows for partial differentiation of HuH7-NTCP and HuH7.5-NTCP cells into hepatocyte-like cells with dHuH7.5-NTCP cells having a stronger hepatocyte phenotype than dHuH7-NTCP cells.

To analyze multi-infections with HBV, HCV, HDV and HEV, we developed a multiplex RT-ddPCR (reverse-transcription droplet digital PCR) assay that allows for the specific and absolute quantification of each of the viral RNAs in a single assay (Fig. S3). Differentiated HuH7-NTCP, dHuH7.5-NTCP, and dHepaRG cells were inoculated with HBV, HDV, HCV, HEV or with the four viruses simultaneously, and levels of intracellular viral RNAs were assessed by multiplex RT-ddPCR at different time points post-inoculation (Fig. 2A). HDV inoculation led to strong replication in the three different cell lines in both mono- or multi-infection settings with a usual peak of replication around 6-9 days post-inoculation. HCV inoculation also led to

representative experiment are presented. (D) 24 h before analysis, media were replaced by serum-free media. Supernatants were collected and ApoB was assessed by ELISA in each fraction of iodixanol gradients. Results from a representative experiment are presented. (E) Cells were stimulated or not with the indicated molecules for 24 h. Total RNAs were extracted, and gene expression was assessed by reverse-transcription quantitative PCR. Levels of target mRNAs were normalized to the levels of Gus-B mRNAs and the no stimulation conditions. Data are the mean of three independent experiments each performed with three biological replicates. dHepaRG, differentiated HepaRG; PHHs, primary human hepatocytes.

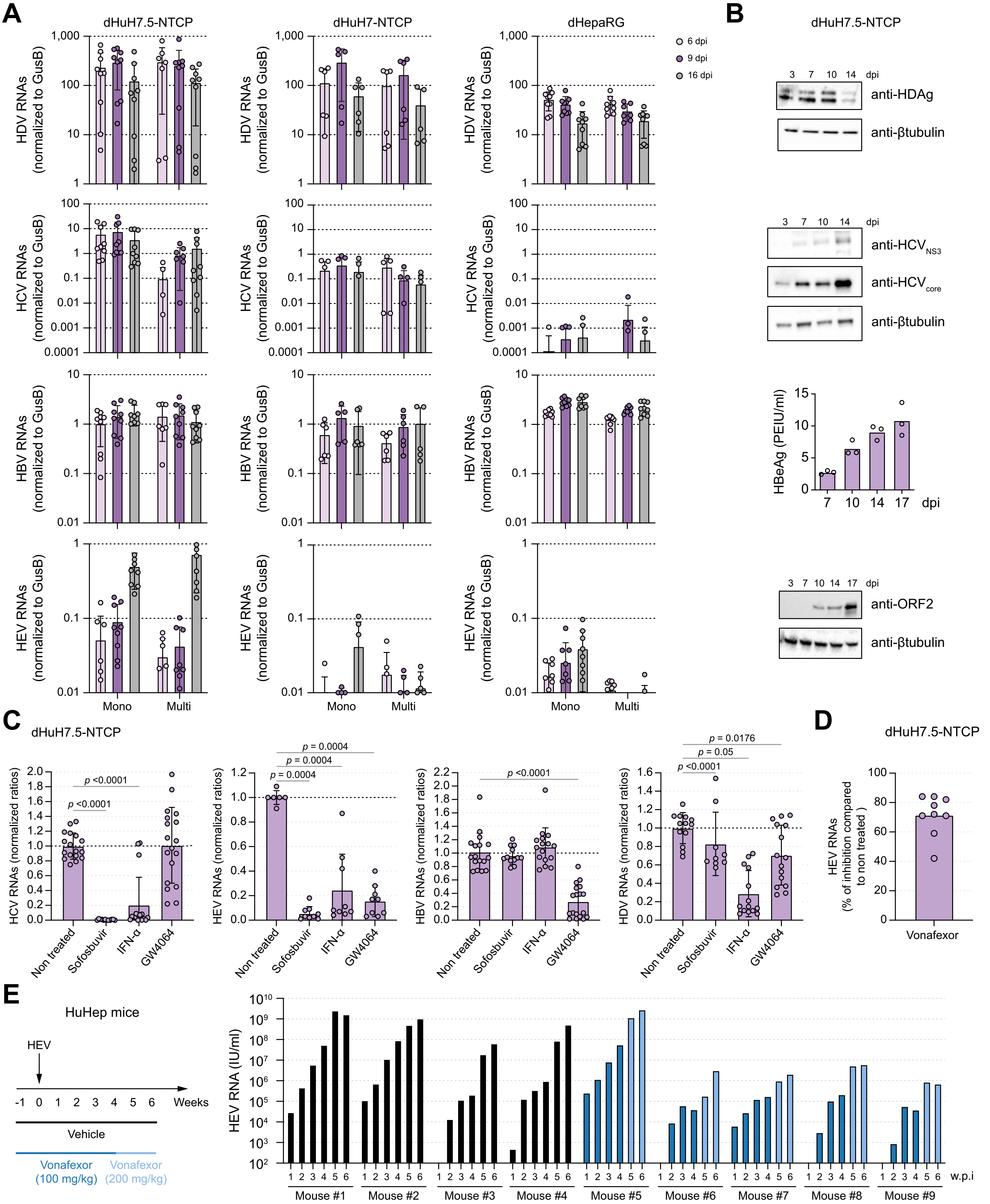

Fig. 2. Infections of dHuH7.5-NTCP cells by HBV, HCV, HDV and HEV and testing for broadly acting antivirals. (A) dHuH7-NTCP, dHuH7.5-NTCP, or dHepaRG cells were inoculated with HBV, HCV, HDV, or HEV (mono) or with the four viruses at the same time (multi). At the indicated dpi, cells were lysed and the levels of viral RNAs were assessed by multiplex RT-ddPCR. (B) dHuH7.5-NTCP cells were inoculated with HCV, HDV, HEV, or HBV. At the indicated dpi, cells were lysed and levels of intracellular viral proteins were analyzed by western blot and the levels of HBeAg in the cell supernatants were quantified by CLIA. (C, D) dHuH7.5-NTCP cells were

potent replication in dHuH7-NTCP and dHuH7.5-NTCP cells, but not in dHepaRG cells in both mono- or multi-infection settings. It should be noted that multi-infection of dHuH7.5-NTCP cells limited HCV replication at day 6 post inoculation, suggesting a possible negative interference of the other viruses on HCV replication at an early time point. HBV inoculation led to comparable HBV RNAs levels in all the tested conditions and cell types. Within the time frame of the experiment, production of HEV RNAs were only observed in dHuH7.5-NTCP cells at day 16 post-inoculation with quasi-enveloped HEV particles (Fig. 2A). These data suggested that only dHuH7.5-NTCP cells can support an efficient replication of the four viruses in mono-infection or co-infection settings. Additional experiments at later time points confirmed persistent replication of HBV, HCV, HDV and HEV in dHuH7.5-NTCP cells in mono-infection (Fig. S4). We also detected viral antigen production by western blot and CLIA (chemiluminescence immunoassay) analyses after mono-infection of dHuH7.5-NTCP cells (Fig. 2B). Of note, double or triple combinations of infection with HBV, HCV, HDV and HEV could also be performed in dHuH7.5-NTCP cells (Fig. S3B) and the interferon-independent viral interference of HDV on HBV we already described[11] could be recapitulated (Fig. S3C). Altogether, our data indicated that dHuH7.5-NTCP cells support mono- and simultaneous multi-infections with HBV, HCV, HDV and HEV and it is likely that dHuH7.5-NTCP cells may also allow for super-infections. However, it remains to be determined if the four viruses can replicate in the same cell or whether exclusion/interference mechanisms exist as suggested for HCV (Fig. 2A). Future single-cell analyses may help to resolve this important question.

To determine if dHuH7.5-NTCP cells are suitable to screen for broadly acting antiviral molecules, we tested three known antivirals in mono-infection settings (Fig. 2C). As expected, sofosbuvir, a nucleoside analogue used to treat HCV-infected patients[30] abrogated HCV infection in dHuH7.5-NTCP cells and highly reduced the levels of HEV RNAs[2] (>90% reduction in the levels of intracellular viral RNAs, p <0.0005), without affecting HBV and HDV RNA levels. Moreover, IFN-α treatment following virus inoculation strongly decreased the levels of intracellular HCV, HDV and HEV RNAs (80% reduction in the levels of intracellular viral RNAs, p <0.0005). However, in contrast to what is usually observed in HBV-infected cells,[31,32] IFN-α had no effect on the levels of HBV RNAs in dHuH7.5-NTCP cells. We recently reported that FXR (farnesoid X receptor) ligands, such as GW4064, can inhibit HBV and HDV replication *in vitro*.[32] Herein, we confirmed this result in dHuH7.5-NTCP cells, with no effect on HCV RNA levels. Interestingly, GW4064 strongly decreased the levels of HEV RNAs by up to 85% (p <0.0001) in dHuH7.5-NTCP cells infected with cell culture-derived quasi-enveloped HEV (Fig. 2C) and vonafexor, a clinical candidate currently being tested against HBV and soon against HDV, showed a similar antiviral effect on HEV (Fig. 2D). We confirmed the strong anti-HEV effect of GW4064 using naked particles of HEV P6 Kernow strain to infect dHuH7.5-NTCP cells (>95% reduction in the levels of intracellular viral RNAs, p = 0.0022) (Fig. S5). Finally, we confirmed that treatment of HuHep mice with vonafexor attenuated HEV infection in four treated animals out of five (Fig. 2E). After cessation of therapy, all four infected HuHep mice experienced a rapid increase in fecal HEV RNA (Fig. S6) to the plateaued levels reached by the control non-treated mice.

Discussion

Altogether, we showed here that two hepatocyte-like cell lines, each with its own advantages and disadvantages, can be used to overcome some issues encountered when working with PHHs. While the immortalized dHepaRG cells exhibit a much more accurate profile regarding innate immune and detoxification pathways, their ability to replicate HCV and HEV is limited. In contrast, the transformed dHuH7.5-NTCP cells display impaired innate immune pathways, but likely a more relevant functional hepatocyte-like lipid metabolism and allow for efficient (multi)-infections with HBV, HDV, HCV and HEV. Of note, even if dHuH7.5-NTCP cells will not permit the investigation of innate immune response to viral infection and its role regarding potential viral interference in the case of multi-infections, their differing innate responsiveness compared to dHepaRG cells or PHHs could be a useful tool. For instance, treatment of dHuH7.5-NTCP cells with IFN-α did not result in a reduction in the levels of intracellular HBV RNAs, in contrast to what is usually observed in HBV-infected HepaRG cells and PHHs.[31,32] We excluded a default in the JAK/STAT pathway in dHuH7.5-NTCP since IFN-α treatment reduced the levels of intracellular HCV, HDV and HEV RNAs (Fig. 2C) and induced IFN-stimulated gene (ISG) expression (Fig. 1E). We instead hypothesize that a different set of ISGs might be produced in dHuH7.5-NTCP cells upon IFN-α treatment, which could explain the differential response. Identification of anti-HBV ISGs could therefore be undertaken by differential analysis.

In conclusion, we established a unique *in vitro* hepatocyte culture model supporting simultaneous infection with the four main hepatotropic viruses (HBV, HCV, HDV and HEV) and allowing us to (i) study the direct interplay between those viruses, (ii) investigate the potential cross-reactivity of antivirals in the case of multi-infections and (iii) screen for/investigate broadly acting antivirals. Additionally, our data highlights FXR ligands, particularly the clinical stage candidate vonafexor, as potential broad-acting antivirals against at least three hepatotropic viruses. Further studies in preclinical models are warranted to move forward in clinical trials.

inoculated with HBV, HCV, HDV, or HEV. Three dpi with HBV, HCV, HDV or 10 dpi with HEV, cells were treated or not with the indicated molecules for 10 days. Cells were lysed and the levels of intracellular viral RNAs were assessed by RT-qPCR. Data are the mean ± SD of at least three independent experiments each performed with three biological replicates. (E) HuHep mice were treated as indicated. At the indicated weeks post-infection, levels of HEV RNA were quantified by qRT-PCR in 10% (w/v) stool suspensions of the infected mice. CLIA, chemiluminescence immunoassay; dpi, days post-inoculation; dHepaRG, differentiated HepaRG; dHuH7-NTCP, DMSO-treated HuH7-NTCP; dHuH7.5-NTCP, DMSO-treated HuH7.5-NTCP; qRT-PCR, quantitative reverse-transcription PCR; RT-ddPCR, reverse-transcription droplet digital PCR. Statistical analyses were performed using a Mann-Whitney U test with the prism software.

Affiliations

[1]CIRI, Centre International de Recherche en Infectiologie, Univ Lyon, Inserm, U1111, Université Claude Bernard Lyon 1, CNRS, UMR5308, ENS de Lyon, F-69007, Lyon, France; [2]Université de Strasbourg, Inserm, Institut de Recherche sur les Maladies Virales et Hépatiques UMR_S1110, Strasbourg, France; [3]Laboratory of Liver Infectious Diseases, Department of Diagnostic Sciences, Faculty of Medicine and Health Sciences, Ghent University, Ghent, Belgium; [4]INSERM, U1052, Cancer Research Center of Lyon (CRCL), University of Lyon (UCBL1), CNRS UMR_5286, Centre Léon Bérard, Lyon, France; [5]Centre Léon Bérard (CLB), INSERM, U1032, Lyon, France; [6]Service de chirurgie générale et oncologique, Hôpital Lyon Sud, Hospices Civils de Lyon Et CICLY, EA3738, université Lyon 1, France; [7]German Centre for Infection Research (DZIF), Department for Molecular & Medical Virology, Ruhr University Bochum, 44801 Bochum, Germany; [8]Dept. of Gastroenterology, Hepatology, Infectious DIseases and Endocrinology, Hannover Medical School, Hannover, Germany; [9]Agence Nationale de Sécurité Sanitaire de L'alimentation de L'environnement et du Travail (ANSES), Institut National de Recherche pour L'agriculture L'alimentation et L'environnement (INRAE), École Nationale Vétérinaire d'Alfort (ENVA), UMR Virology, 94700 Maisons-Alfort, France; [10]ENYO Pharma, Lyon, France; [11]German Centre for Infection Research (DZIF), partner-site Hannover-Braunschweig, Excellence Cluster RESIST, D-SOLVE consortium, Germany

Abbreviations

DMSO, dimethyl sulfoxide; dHepaRG, differentiated HepaRG; dHuH7-NTCP, DMSO-treated HuH7-NTCP; dHuH7.5-NTCP, DMSO-treated HuH7.5-NTCP; ISG, IFN-stimulated gene; PHHs, primary human hepatocytes; VLDL, very low-density lipoprotein.

Financial support

This work was supported by several grants from the ANRS MIE (French national agency for research on AIDS, viral hepatitis and emerging diseases, CSS12, ECTZ172540, ANRS0544, ECTZ187893, ECTZ244976), as well as financial support of INSERM and CNRS. RF was supported by PhD scholarships from University Claude Bernard Lyon 1. E.R.V. acknowledges fundings from the French National Research Agency (ANR, grant number ANR-21-CE15-0035-01 DELTArget) and fundings from the interdisciplinary Thematic Institute IMCBio, as part of the ITI 2021-2028 program of the University of Strasbourg, CNRS and Inserm, was supported by IdEx Unistra (ANR-10-IDEX-0002), and by SFRI-STRAT'US project (ANR-20-SFRI-0012) and EUR IMCBio (ANR-17-EURE-0023) under the framework of the French Investments for the Future Program. ES was supported by a grant of the German Centre for Infection Diseases (DZIF). PM was supported by Ghent University (PhD fellowship to ADM, and BOF.-BAF.2024.0637.01) and grants from the Research Foundation-Flanders (FWO-Vlaanderen; Excellence of Science (EOS) project VirEOS2.0 and research project G0A7Y24N).

Conflict of interest

The authors of this study declare that they do not have any conflict of interest.

Please refer to the accompanying ICMJE disclosure forms for further details.

Authors' contributions

Study concept and design: JL, DD, PM; Acquisition of data: RF, MM, CP, ADM, LV; Analyses and interpretation of data: RF, EV, PM, DD, JL; Drafting of the manuscript: RF, EV, DD, JL; Funding acquisition: JL, DD, AS, PM; Material support: OD, RB, VD, NP, ES, GP, MR, RD, PM, HW.

Data availability statement

The data presented in this manuscript are available through the corresponding authors (Julie Lucifora and David Durantel) upon reasonable request. Next-generation sequencing was performed by the Biomedical Sequencing Facility at CeMM Research Center for Molecular Medicine of the Austrian Academy of Sciences. RNA-seq data presented in this manuscript are accessible through Gene Expression Omnibus (GSE288203 and GEO GSE288204).

Acknowledgements

The authors would like to thank Pr Fabien Zoulim, Dr Barbara Testoni and Pr Massimo Levrero for the access to primary human hepatocyte (PHH) isolation platform, as well as Anaëlle Dubois, Sarah Heintz, Isabelle Bordes and Emilie Charles for their help with the isolation of PHH. Moreover, authors would like to thank Prof Michel Rivoire's and Dr Guillaume Passot's, and their respective staff in surgery room, for providing us with liver resections. We also thank the BSF team at CeMM Research Center for Molecular Medicine of the Austrian Academy of Sciences (Vienna, AUT) as well as our colleague Dr Frank Jühling (U1110) for the RNAseq analyses.

Supplementary data

Supplementary data to this article can be found online at https://doi.org/10.1016/j.jhepr.2025.101383.

References

Author names in bold designate shared co-first authorship

[1] Lanini S, Ustianowski A, Pisapia R, et al. Viral hepatitis: etiology, epidemiology, transmission, diagnostics, treatment, and prevention. Infect Dis Clin North Am 2019;33:1045–1062.
[2] **Kinast V**, **Burkard TL**, Todt D, et al. Hepatitis E virus drug development. Viruses 2019;11.
[3] Riaz M, Idrees M, Kanwal H, et al. An overview of triple infection with hepatitis B, C and D viruses. Virol J 2011;8:368.
[4] Takahashi M, Nishizawa T, Gotanda Y, et al. High prevalence of antibodies to hepatitis A and E viruses and viremia of hepatitis B, C, and D viruses among apparently healthy populations in Mongolia. Clin Diagn Lab Immunol 2004;11:392–398.
[5] Mazhar MKA, Finger F, Evers ES, et al. An outbreak of acute jaundice syndrome (AJS) among the Rohingya refugees in Cox's Bazar, Bangladesh: findings from enhanced epidemiological surveillance. PLoS One 2021;16:e0250505.
[6] Makiala-Mandanda S, Le Gal F, Ngwaka-Matsung N, et al. High prevalence and diversity of hepatitis viruses in suspected cases of yellow fever in the democratic republic of Congo. J Clin Microbiol 2017;55:1299–1312.
[7] Wu JC, Chen CL, Hou MC, et al. Multiple viral infection as the most common cause of fulminant and subfulminant viral hepatitis in an area endemic for hepatitis B: application and limitations of the polymerase chain reaction. Hepatology 1994;19:836–840.
[8] Weltman MD, Brotodihardjo A, Crewe EB, et al. Coinfection with hepatitis B and C or B, C and delta viruses results in severe chronic liver disease and responds poorly to interferon-alpha treatment. J Viral Hepat 1995;2:39–45.
[9] Nasir M, Wu GY. HEV and HBV dual infection: a review. J Clin Transl Hepatol 2020;8:313–321.
[10] Lutterkort GL, Wranke A, Hengst J, et al. Viral dominance patterns in chronic hepatitis delta determine early response to interferon alpha therapy. J Viral Hepat 2018;25:1384–1394.
[11] Lucifora J, Alfaiate D, Pons C, et al. Hepatitis D virus interferes with hepatitis B virus RNA production via interferon-dependent and -independent mechanisms. J Hepatol 2023;136:19–31.
[12] **Burkard T**, **Proske N**, Resner K, et al. Viral interference of hepatitis C and E virus replication in novel experimental Co-infection systems. Cells 2022;11.
[13] Liaw YF, Tsai SL, Sheen IS, et al. Clinical and virological course of chronic hepatitis B virus infection with hepatitis C and D virus markers. Am J Gastroenterol 1998;93:354–359.
[14] Bellecave P, Gouttenoire J, Gajer M, et al. Hepatitis B and C virus coinfection: a novel model system reveals the absence of direct viral interference. Hepatology 2009;50:46–55.
[15] Deterding K, Pothakamuri SV, Schlaphoff V, et al. Clearance of chronic HCV infection during acute delta hepatitis. Infection 2009;37:159–162.
[16] Kilonzo SB, Wang YL, Jiang QQ, et al. Superinfective hepatitis E virus infection aggravates hepatocytes injury in chronic hepatitis B. Curr Med Sci 2019;39:719–726.
[17] Schulz M, Schott E. An unusual cause for a hepatic flare in a chronic HBV carrier. Hepat Mon 2014;14:e20099.
[18] Heuschkel MJ, Baumert TF, Verrier ER. Cell culture models for the study of hepatitis D virus entry and infection. Viruses 2021;13.

[19] **Todt D**, **Friesland M**, Moeller N, et al. Robust hepatitis E virus infection and transcriptional response in human hepatocytes. Proc Natl Acad Sci U S A 2020;117:1731–1741.
[20] Alfaiate D, Lucifora J, Abeywickrama-Samarakoon N, et al. HDV RNA replication is associated with HBV repression and interferon-stimulated genes induction in super-infected hepatocytes. Antivir Res 2016;136:19–31.
[21] Pellerin M, Hirchaud E, Blanchard Y, et al. Characterization of a cell culture system of persistent hepatitis E virus infection in the human HepaRG hepatic cell line. Viruses 2021;13.
[22] Sumpter Jr R, Loo YM, Foy E, et al. Regulating intracellular antiviral defense and permissiveness to hepatitis C virus RNA replication through a cellular RNA helicase, RIG-I. J Virol 2005;79:2689–2699.
[23] Devhare PB, Desai S, Lole KS. Innate immune responses in human hepatocyte-derived cell lines alter genotype 1 hepatitis E virus replication efficiencies. Scientific Rep 2016;6:26827.
[24] **Yan H**, **Zhong G**, Xu G, et al. Sodium taurocholate cotransporting polypeptide is a functional receptor for human hepatitis B and D virus. eLife 2012;1:e00049.
[25] Sainz Jr B, Chisari FV. Production of infectious hepatitis C virus by well-differentiated, growth-arrested human hepatoma-derived cells. J Virol 2006;80:10253–10257.
[26] Quasdorff M, Protzer U. Control of hepatitis B virus at the level of transcription. J Viral Hepat 2010;17:527–536.
[27] Delphin M, Desmares M, Schuehle S, et al. How to get away with liver innate immunity? A viruses' tale. Liver Int 2021;41:2547–2559.
[28] **Luangsay S**, **Ait-Goughoulte M**, Michelet M, et al. Expression and functionality of Toll- and RIG-like receptors in HepaRG cells. J Hepatol 2015;63:1077–1085.
[29] Tang ED, Wang CY. TRAF5 is a downstream target of MAVS in antiviral innate immune signaling. PLoS One 2010;5:e9172.
[30] Lee R, Kottilil S, Wilson E. Sofosbuvir/velpatasvir: a pangenotypic drug to simplify HCV therapy. Hepatol Int 2017;11:161–170.
[31] **Michelet M**, **Alfaiate D**, **Chardes B**, et al. Inducers of the NF-kappaB pathways impair hepatitis delta virus replication and strongly decrease progeny infectivity in vitro. JHEP Rep 2022;4:100415.
[32] **Legrand AF**, **Lucifora J**, Lacombe B, et al. Farnesoid X receptor alpha ligands inhibit HDV in vitro replication and virion infectivity. Hepatol Commun 2023;7.

Keywords: Hepatitis B Virus; Hepatitis D Virus; Hepatitis C Virus; Hepatitis E Virus; hepatocytes; drug screening; broad acting antivirals.

Received 20 November 2024; received in revised form 21 February 2025; accepted 25 February 2025; Available online 28 February 2025

Journal of Hepatology, Volume *7*

Supplemental information

A novel *in vitro* system for simultaneous infections with hepatitis B, C, D and E viruses

Roxanne Fouillé, Eloi R. Verrier, Amse De Meyer, Lieven Verhoye, Maud Michelet, Romain Barnault, Caroline Pons, Olivier Diaz, Michel Rivoire, Guillaume Passot, Eike Steinmann, Heiner Wedemeyer, Anna Salvetti, Nicole Pavio, Virginie Doceul, Raphaël Darteil, Philip Meuleman, David Durantel, and Julie Lucifora

A novel *in vitro* system for simultaneous infections with hepatitis B, C, D and E viruses

Roxanne Fouillé, Eloi R. Verrier, Amse De Meyer, Lieven Verhoye, Maud Michelet, Romain Barnault, Caroline Pons, Olivier Diaz, Michel Rivoire, Guillaume Passot, Eike Steinmann, Heiner Wedemeyer, Anna Salvetti, Nicole Pavio, Virginie Doceul, Raphaël Darteil, Philip Meuleman, David Durantel, Julie Lucifora

Table of contents

Materials and methods……………………………………………………………………2

Supplementary figures………………………………………………………………………5

Original western blots……………………………………………………………………11

Supplementary tables……………………………………………………………..…………12

Supplementary references…………………………………………………………………...18

Materials and methods

Cell culture and viral infection. HepaRG cells were cultured and differentiated as previously described [1, 2]. Primary human hepatocytes (PHH) were isolated from human liver resections obtained from the Centre Léon Bérard (Lyon) and Hopital de Lyon Sud with French ministerial authorizations (AC 2013-1871, DC 2013 – 1870, AFNOR NF 96 900 sept 2011) as previously described [3]. The HuH7-NTCP and HuH7.5-NTCP are cultured in 10% FCS-supplemented DMEM and differentiated with 2% of DMSO for 1 week without splitting the cells. HBV (genotype D) inocula were prepared from HepAD38 supernatants [4] and used with a multiplicity of infection (m.o.i) of 1000 vge/cells. HDV inocula (genotype 1) were prepared from supernatants from HuH7-2C8D as previously described [5] and used with a m.o.i of 500 vge/cells. Viral stocks of HCV genotype 2A JFH1 strain were generated as previously described [6] and used with a m.o.i of 0,2 vge/cells. Quasi-enveloped HEV-3 was produced by infection of dHepaRG cells and collection of supernatant as previously described [7] and used with a m.o.i of 10 vge/cells. Alternatively, intracellular cell culture HEV-3 p6 Kernow (naked virus) was also used in Figure S4 and S5 [8]. Infections were performed overnight with 4% PEG.

Reagents. TLR1/2 ligand (Pam3CSK4, used at 10 ug/mL), TRL3-L (Riboxxol, used at 10 ug/mL), TLR4-L (LPS, used at 10 ug/mL), RLR-L (transfected polyI:C, used at 1 ug/mL) or IFN-α (used at 500 or 1000 IU/mL). Pam3CSK4, LPS and PolyIC HMW were purchased from Invivogen. Riboxxol was purchased from Ribox and IFN-α (Roferon, used at 500 IU/mL) was purchased from Roche. Sofosbuvir (used at 10 µM) and GW4064 (used at 10 µM) were purchased from Selleckchem, RG7834 (used at 10 µM) was synthetized by AI-Biopharma. Vonafexor, provided by ENYO Pharma, was used at 10 µM.

Protein expression analysis. For western blot analyses, cells were lyzed with a RIPA lysis buffer (Tris-HCl pH 7,5 10mM, NaCl 140mM, EDTA 1mM, EGTA 0,5mM, 1% Triton X100, 0,1% SDS, 0,1% Na-Deoxycholate) containing protease and phosphatase inhibitors (Roche). We used the following primary antibodies: anti-γ-tubulin (Sigma, T6557), anti-albumin (Dako), anti-ORF2 (Milipore MAB8002), anti-NS3 (Virogen 217-A), anti-HCV core (Thermofisher MA1-7366). The anti-HDAg, a polyclonal rabbit antibody was produced by Eurogentec for in-house use. HBsAg were detected in the supernatant of HBV-infected cells using the Autobio kit according to the manufacturer (AutoBio, China)

ApoB secretion and VLDL analyses. To assess ApoB containing VLDL production, cell supernatants were loaded on a 7-56 % iodixanol gradient as previously described [9]. After an overnight ultracentrifugation, fractions were collected and levels of ApoB were quantified by ELISA in each fraction using anti-ApoB (Biodesign H45640M) and anti-ApoB conjugated with HRP (Biodesign K34005G).

Nucleic acids detection. Total intracellular RNAs were extracted with the "Monarch, nucleic acid purification kit" according to the manufacturer's instructions (New England Biolabs). RNA reverse transcription assays were performed using the LunaScript RT Super Mix (New England BioLabs). Quantitative PCR was performed using specific primers (Table 1) and normalized to the household gene Gus-B using the Luna Universal qPCR Master Mix (New England BioLabs). To detect multi-infections, we set up a multiplex RT-ddPCR assay using the "ddPCR Multiplex Supermix" (Bio-Rad) according to the manufacturer's instruction with specific primers (0,9 uM final) and probes (0,25 uM final) (Table S1). Droplets were generated using and the" QX200™ Droplet Generator" (Bio-Rad) and analyzed after PCR with the "QX600 Droplet Reader" (Bio-Rad).

HuHep mice experiment. Human-liver chimeric mice (HuHep mice) were generated by transplanting 10^6 primary human hepatocytes (Lonza, Basel, Switzerland; donor HUM191501) into the spleen of uPA$^{+/+}$-SCID mice. Successful humanization was confirmed by quantification of human albumin in mouse plasma using a human-specific ELISA (Bethyl Laboratories, USA). Mice with human albumin levels ranging between 3-16 mg/mL were used in this study. For *in vivo* prevention of HEV infection,

HuHep mice (n=6) received a 7-week Vonafexor treatment starting 7 days before HEV inoculation (intraperitoneal injection of a filtered stool suspension containing 10^4 IU of a mouse-passaged genotype 3 patient isolate). Vonafexor was formulated as a suspension (15 and 25 mg/ml) in deionized water supplemented with 0.5% carboxymethylcellulose and 0.25% Tween80, and was administered daily via oral gavage. Vonafexor was dosed at 100 mg/kg (15 mg/ml) until 4 weeks post-infection, after which the dose was increased to 200 mg/kg (25 mg/ml). Two weeks later treatment was stopped. Control animals were treated with vehicle only, according to the same regimen as Vonafexor. Stool samples were collected weekly and HEV RNA content was analyzed in 10% (w/v) suspensions as described before [10]. Briefly, total RNA was extracted from a 10% (w/v) stool suspension with the NucliSENS easyMAG device according to the manufacturer's instructions (Biomérieux). Quantitative PCR was performed on the LightCycler 480 (Roche Diagnostics) by using the LightCycler Multiplex RNA Virus Master mix (Roche) and specific primers (5'-GGTGGTTTCTGGGGTGAC-3' and 3'-AGGGGTTGGTTGGATGAA-5') and probe (5'-FAM-TGATTCTCAGCCCTTCGC-TAMRA-3'). Quantification was performed by using an in-house standard curve based on the WHO 1st international standard of HEV (Paul-Ehrlich Institute). All mice were bred under sterile conditions and all experiments were approved by the Animal Ethics Committee of the Faculty of Medicine and Health Sciences of Ghent University (ref. ECD 22/46)

Statistical analyses. Statistical were performed using a Mann-Whitney U test with the prism software. p values are indicated directly in the graphs.

Next-Generation Sequencing and Raw Data Acquisition. Two RNA-seq datasets were produced and are accessible through Gene Expression Omnibus (GSE288204 and GSE288203). Expression profiling libraries were prepared using NEBNext Ultra II Directional RNA with UMI Adaptors kit (New England Biolabs) and Stranded mRNA kit (Illumina) for GEO DIF and GEO IFNa, respectively. Libraries were sequenced on a HiSeq 3000 (GSE288204) or a HiSeq 4000 (GSE288203) instrument (Illumina, San Diego, CA, USA) following a 50-base-pair, single-end recipe. Raw data acquisition (HiSeq Control Software, HCS, HD 3.4.0.38) and base calling (Real-Time Analysis Software, RTA, 2.7.7) was performed on-instrument, while the subsequent raw data processing off the instruments involved two custom programs based on Picard tools (2.19.2). In a first step, base calls were converted into lane-specific, multiplexed, una-ligned BAM files suitable for long-term archival (IlluminaBasecallsToMultiplexSam, 2.19.2-CeMM). In a second step, archive BAM files were demultiplexed into sample-specific, unaligned BAM files (Illumi-naSamDemux, 2.19.2-CeMM).

Transcriptome Analysis. NGS reads were mapped to the Genome Reference Consortium GRCh38 assembly via "Spliced Transcripts Alignment to a Reference" (STAR, 2.7.5a) utilizing the "basic" ensembl transcript annotation from version e100 (April 2020) as reference transcriptome. Since the hg38 assembly flavour of the UCSC Genome Browser was preferred for downstream data processing with Bioconductor packages for entirely technical reasons, ensembl transcript annotation had to be adjusted to UCSC Genome Browser sequence region names. STAR was run with options recommended by the ENCODE project. NGS read alignments overlapping ensembl transcript features were counted with the Bioconductor (3.12 and 3.11) Genomic Alignments (1.26.0 and 1.24.0) package (GSE288204 and GSE288203, respectively). Transcript-level counts were aggregated to gene-level counts and the Bioconductor DESeq2 (1.30.0 for GEO DIF and 1.28.1 for GSE288203) package was used to test for differential expression based on a model using the negative binomial distribution. Pathway analysis was performed through Gene Set Enrichment Analysis (GSEA) [11]. Regarding GSE288204, replicate number 2 of differentiated Huh7-NTCP cells was considered as an outlier and removed from the analysis. The expression of 263 liver-specific genes according the Human Protein Atlas [12] was investigated
(https://www.proteinatlas.org/humanproteome/tissue/liver#the_liver_specific_proteome, accessed online on November 4, 2024). Excluding the non-expressed genes in the cell lines, the expression of

250 and 243 genes are presented for HuH7.5-NTCP and HuH7-NTCP cells (Figure 1B and Table S2), respectively, using the Z-score transformation as already published [13].

Supplementary figures

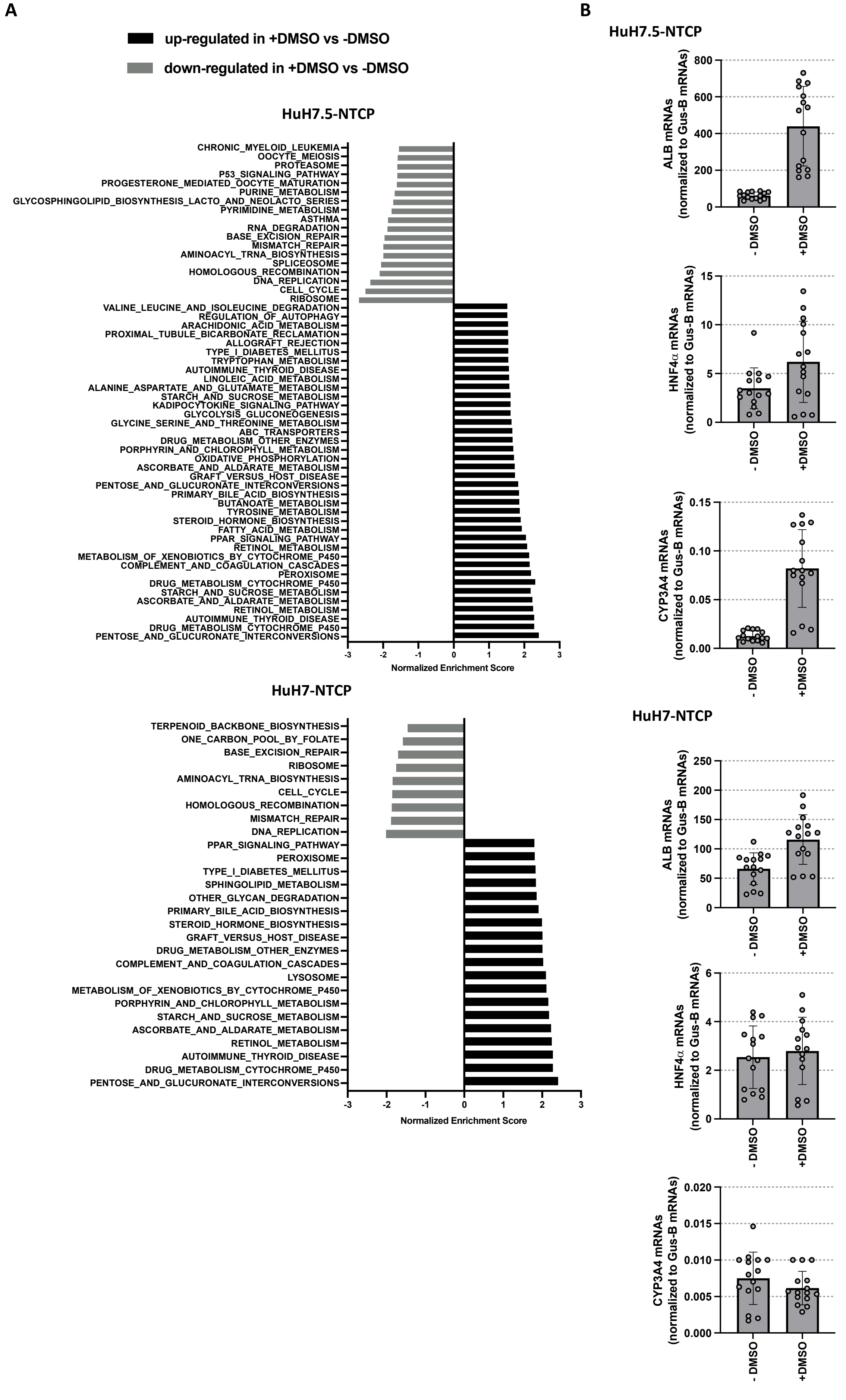

Fig. S1: DMSO treatment of HuH7.5-NTCP and HuH7-NTCP cells allows expression of liver-specific genes. (A) HuH7-NTCP or HuH7.5-NTCP cells have been seeded and treated or not with 2% DMSO as indicated in Figure 1A before analyses. Cells were lysed and total RNAs were analyzed by RNA sequencing. Pathway analyses were performed with KEGG and are presented for each cell line as normalized enrichment score. (B) Cells were lysed and total RNAs were analyzed by specific RT-qPCR analyses. Levels of target mRNAs were normalized to the levels of Gus-B mRNAs. Data are the mean +/-SD of 3 to 5 independent experiments each performed with 2 or 3 biological replicates. Dots represents the different biological replicates from all the experiments.

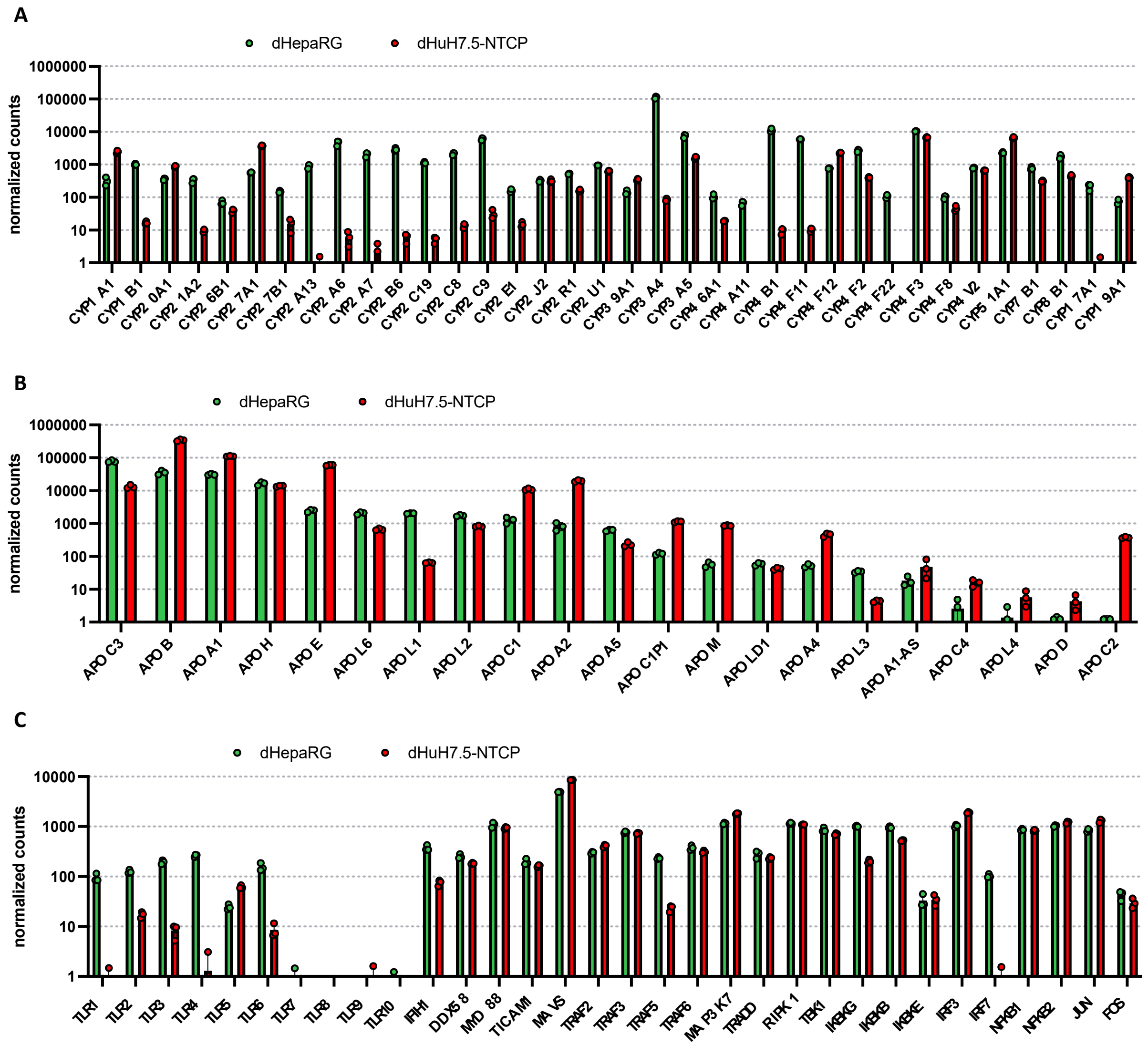

Fig. S2: Comparison of gene expression in dHepaRG and dHuH7.5-NTCP cells. **T**otal RNAs extracted from dHepaRG or dHuH7.5-NTCP cells were analyzed by RNA sequencing. Normalized counts for (A) cytochrome related, (B) apolipoprotein-related or (C) RLR pathway related transcript are presented. Results are the mean +/- SD of 3 biological replicates. Dots represents the different biological replicates.

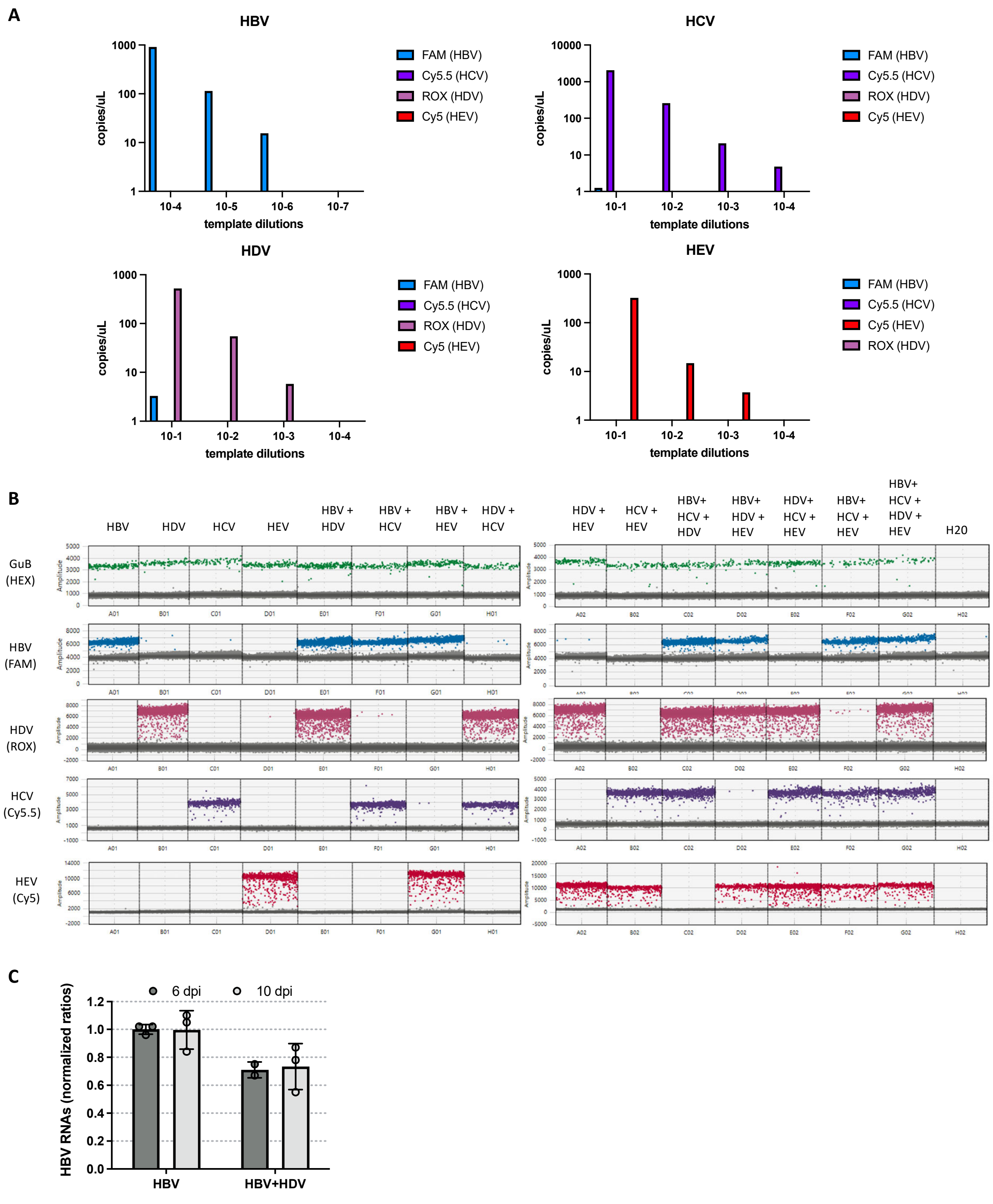

Fig. S3: Multiplex RT-ddPCR assay for simultaneous detection of HBV, HCV, HDV and HEV. (A) To determine the specificity of the assay, total nucleic acids from HBV, HCV, HEV and HDV inocula were extracted and RT-ddPCR analyses were performed with serial dilutions of each DNA/cDNA and a ddPCR reaction mix containing the 4 different sets of primers and probes to detect the 4 viral nucleic acids. (B) dHuH7.5-NTCP were inoculated with HCV, HDV, HEV, HBV or different combination of the 4 viruses at the same time. At days 10 post-inoculation, cells were lysed and the levels of viral RNAs were assessed by multiplex RT-ddPCR with specific probes. (C) dHuH7.5-NTCP were inoculated with HBV or HBV+HDV. At the indicated day post-inoculation, cells were lysed and the levels of HBV RNAs were assessed by multiplex RT-ddPCR with specific probes. Data (normalized to GusB mRNA and the HBV alone condition) are the mean +/-SD one two to three biological replicate.

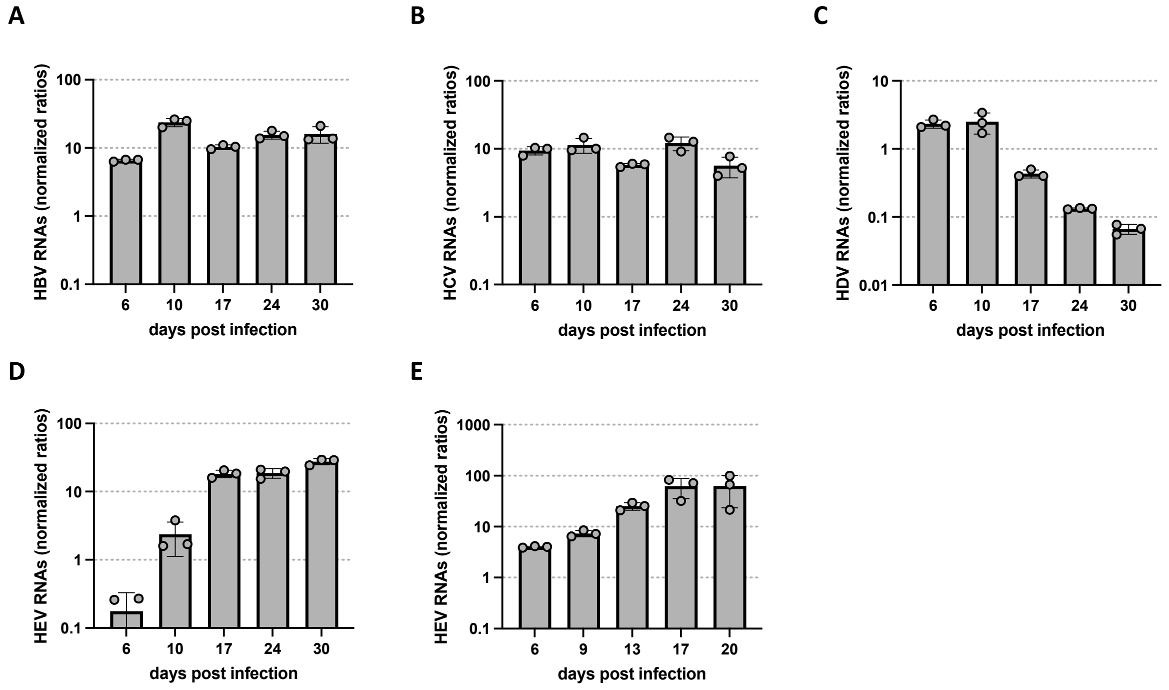

Fig. S4: Infections of dHuH7.5-NTCP cells by HBV, HCV, HDV and different HEV strains. HuH7.5-NTCP were differentiated with 2% DMSO for a week before inoculations with (A) HBV, (B) HCV, (C) HDV, (D) extracellular dHepaRG cells derived HEV-3 i.e. enveloped virus or (E) intracellular cell culture derived p6 HEV-3 i.e. naked virus. At the indicated days post-infection cells were lysed and the levels of intracellular viral RNAs were assessed by RT-qPCR. Data are the mean +/- SD of three biological replicate. Dots represents the different biological replicates.

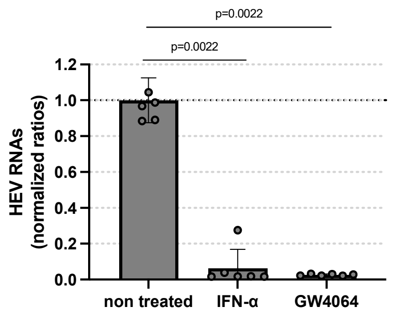

Fig. S5: Treatments with IFN-a or FXR-ligands abrogate HEV infection in dHuH7.5-NTCP cells. HuH7.5-NTCP were differentiated with 2% DMSO for a week before inoculations with intracellular cell culture derived p6 HEV-3 i.e. naked virus. Three days post-inoculation, cells were treated or not with IFN-α (500 IU/mL) or GW4064 (10 µM) for 10 days. Cells were lysed and the levels of intracellular HEV RNAs were assessed by RT-qPCR. Data are the mean +/- SD of two independent experiments each performed with three biological replicate. Dots represents the different biological replicates from all the experiments.

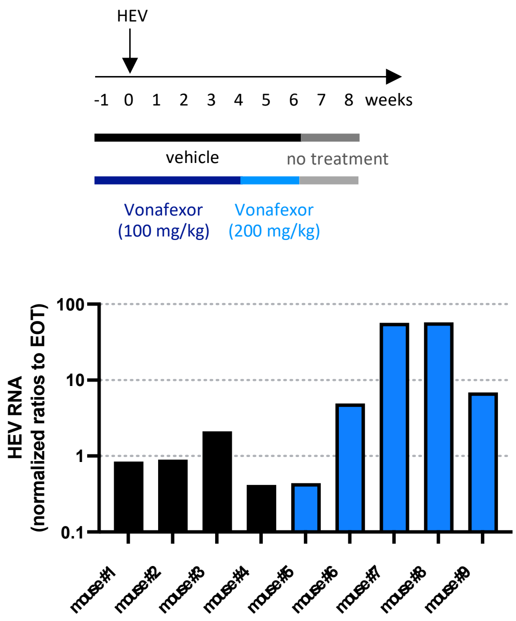

Fig. S6: Viral kinetics after cessation of Vonafexor treatment. HuHep mice were treated and infected with HEV-3 as indicated. Two weeks after arrest of treatment (8 weeks post-infection), levels of HEV RNAs in the stool of mice were quantified by qRT-PCR and normalized to levels at end of treatment (EOT).

Original Western blots

Figure 1C

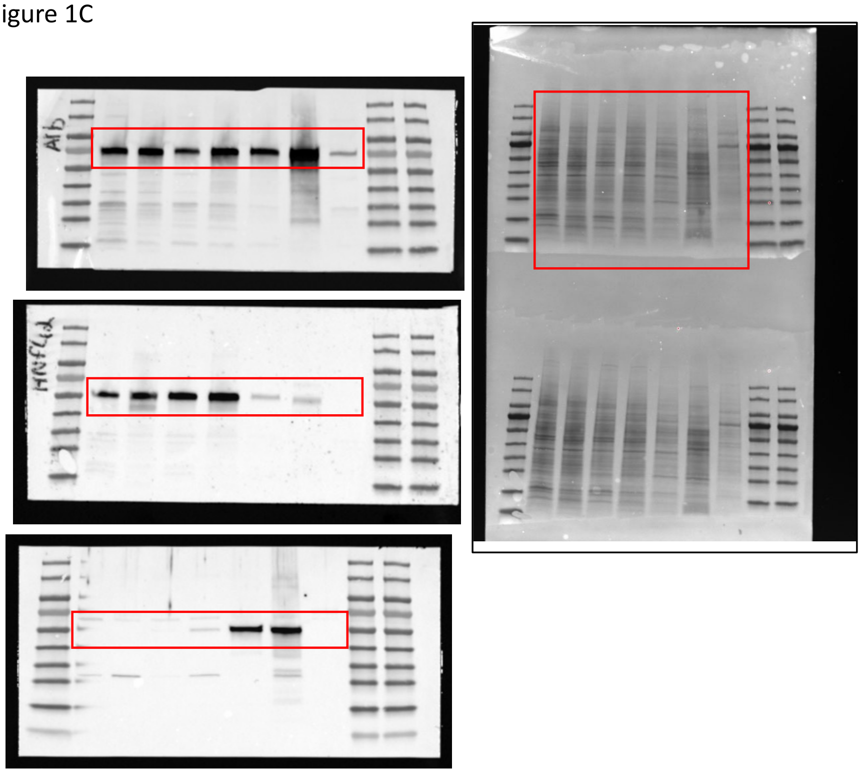

Figure 2B

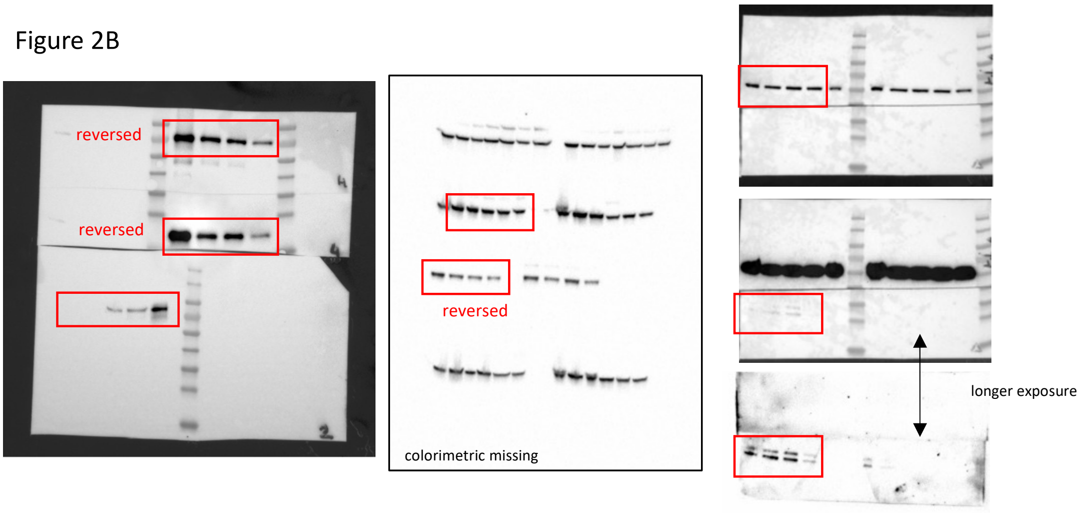

Supplementary tables

mRNAs	Forward primer sequence (5'-3')	Reverse primer sequence (3'-5')	Probe for ddPCR
Albumin	CTGCACAGAATCCTTGGTGAAC	TTTGGGAACGTATGTTTCATCG	
CYP3A4	CTTCATCCAATGGACTGCATAAAT	TCCCAAGTATAACACTCTACACAGACAA	
HNF4α	GAGTGGGCCAAGTACA	GGCTTTGAGGTAGGCATA	
RSAD2	CTTTGTGCTGCCCCTTGAG	TCCATACCAGCTTCCTTAAGCAA	
IL-6	TCGAGCCCACCGGGAACGAA	GCAACTGGACCGAAGGCGCT	
HBV	ACCGAATGTTGCCCAAGGTC	TATGCCTCAAGGTCGGTCGT	[FAM]-TCAACGACCGACCTTGAGGCA[BHQ1]
HDV	CGGGCCGGCTACTCTTCT	AAGGAAGGCCCTCGAGAACA	[ROX]-TGCCTCCCGCCGATAGCTGCT-[BHQ2]
HCV	CTCCCGGGGCACTCGCAAGC	GTCTAGCCATGGCGTTAGTA	[Cy5.5]-GCCTCCAGGCCCCCCCCTCC-[BHQ3]
HEV (quasi-enveloped)	GGTGGTTTCTGGGGTGAC	AGGGGTTGGTTGGATGAA	[Cy5]TGATTCTCAGCCCTTCGC-[BHQ2]
Gus B	CGTGGTTGGAGAGCTCATTTGGAA	ATTCCCCAGCACTCTCGTCGGT	[HEX]-CGTGTCCCTTCCTCCCCGAG-IB(R)FQ
HEV (naked)	ATTGGCCAGAAGTTGGTTTTCAC	CCGTGGCTATAATTGTGGTCT	

Table S1. Primers and probes for qPCR or ddPCR analyses.

**Gene Name**	**Ensembl**	**Uniprot**	**Expressed in HuH7.5-NTCP cells**	**Expressed in HuH7-NTCP cells**
A1BG	ENSG00000121410	P04217	X	X
A1CF	ENSG00000148584	Q9NQ94	X	X
ABCB11	ENSG00000073734	O95342	X	X
ABCB4	ENSG00000005471	P21439	X	X
ACADSB	ENSG00000196177	P45954	X	X
ACAT2	ENSG00000120437	Q9BWD1	X	X
ACOT12	ENSG00000172497	Q8WYK0	X	X
ACOX2	ENSG00000168306	Q99424	X	X
ACSM5	ENSG00000183549	Q6NUN0	X	X
ADH1A	ENSG00000187758	P07327	X	X
ADH4	ENSG00000198099	P08319	X	X
ADH6	ENSG00000172955	P28332	X	X
AFM	ENSG00000079557	P43652	X	X
AFP	ENSG00000081051	P02771	X	X
AGMO	ENSG00000187546	Q6ZNB7	X	X
AGT	ENSG00000135744	P01019	X	X
AGXT	ENSG00000172482	P21549	X	X
AHSG	ENSG00000145192	P02765	X	X
AKR1C4	ENSG00000198610	P17516	X	X
AKR1D1	ENSG00000122787	P51857	X	X
ALB	ENSG00000163631	P02768	X	X
AMBP	ENSG00000106927	P02760	X	X
ANG	ENSG00000214274	P03950	X	X
ANGPTL3	ENSG00000132855	Q9Y5C1	X	X
ANGPTL6	ENSG00000130812	Q8NI99	X	X
ANGPTL8	ENSG00000130173	Q6UXH0	X	X
APCS	ENSG00000132703	P02743	X	X

APOA1	ENSG00000118137	P02647	X	X
APOA2	ENSG00000158874	P02652	X	X
APOA5	ENSG00000110243	Q6Q788	X	X
APOC1	ENSG00000130208	P02654	X	X
APOC2	ENSG00000234906	P02655	X	X
APOC3	ENSG00000110245	P02656	X	X
APOC4	ENSG00000267467	P55056	X	X
APOC4-APOC2	ENSG00000224916		No	No
APOF	ENSG00000175336	Q13790	X	X
APOH	ENSG00000091583	P02749	X	X
APOM	ENSG00000204444	O95445	X	X
AQP9	ENSG00000103569	O43315	No	No
ARG1	ENSG00000118520	P05089	X	X
ARID3C	ENSG00000205143	A6NKF2	X	X
ARMC6	ENSG00000105676	Q6NXE6	X	X
ASGR1	ENSG00000141505	P07306	X	X
ASGR2	ENSG00000161944	P07307	X	X
ASL	ENSG00000126522	P04424	X	X
ATF5	ENSG00000169136	Q9Y2D1	X	X
BAAT	ENSG00000136881	Q14032	X	X
BCHE	ENSG00000114200	P06276	X	X
BDH1	ENSG00000161267	Q02338	X	X
C1S	ENSG00000182326	P09871	X	X
C2	ENSG00000166278	P06681	X	X
C3	ENSG00000125730	P01024	X	X
C4BPA	ENSG00000123838	P04003	X	X
C4BPB	ENSG00000123843	P20851	X	X
C5	ENSG00000106804	P01031	X	X
C6	ENSG00000039537	P13671	X	X
C8A	ENSG00000157131	P07357	X	X
C8B	ENSG00000021852	P07358	X	X
C8G	ENSG00000176919	P07360	X	X
C9	ENSG00000113600	P02748	X	X
CA5A	ENSG00000174990	P35218	X	X
CCDC152	ENSG00000198865	Q4G0S7	X	X
CCL16	ENSG00000275152	O15467	X	X
CES1	ENSG00000198848	P23141	X	X
CFB	ENSG00000243649	P00751	X	X
CFH	ENSG00000000971	P08603	X	X
CFHR1	ENSG00000244414	Q03591	X	X
CFHR2	ENSG00000080910	P36980	X	X
CFHR3	ENSG00000116785	Q02985	X	X
CFHR4	ENSG00000134365	Q92496	X	X
CFHR5	ENSG00000134389	Q9BXR6	X	X
CFI	ENSG00000205403	P05156	X	X
CLEC1B	ENSG00000165682	Q9P126	X	No
COLEC10	ENSG00000184374	Q9Y6Z7	X	X
CP	ENSG00000047457	P00450	X	X
CPB2	ENSG00000080618	Q96IY4	X	X
CPN1	ENSG00000120054	P15169	X	X
CPN2	ENSG00000178772	P22792	X	X

CPS1	ENSG00000021826	P31327	X	X
CRP	ENSG00000132693	P02741	X	X
CXCL2	ENSG00000081041	P19875	X	X
CYP1A2	ENSG00000140505	P05177	X	X
CYP26A1	ENSG00000095596	O43174	X	X
CYP2A13	ENSG00000197838	Q16696	X	X
CYP2A6	ENSG00000255974	P11509	X	X
CYP2A7	ENSG00000198077	P20853	X	X
CYP2B6	ENSG00000197408	P20813	X	X
CYP2C19	ENSG00000165841	P33261	X	X
CYP2C8	ENSG00000138115	P10632	X	X
CYP2C9	ENSG00000138109	P11712	X	X
CYP2D6	ENSG00000100197	P10635	X	X
CYP2E1	ENSG00000130649	P05181	X	X
CYP39A1	ENSG00000146233	Q9NYL5	X	X
CYP3A4	ENSG00000160868	P08684	X	X
CYP3A43	ENSG00000021461	Q9HB55	X	X
CYP3A7	ENSG00000160870	P24462	X	X
CYP3A7-CYP3A51P	ENSG00000282301		No	No
CYP4A22	ENSG00000162365	Q5TCH4	X	No
CYP7A1	ENSG00000167910	P22680	X	No
CYP8B1	ENSG00000180432	Q9UNU6	X	X
DCXR	ENSG00000169738	Q7Z4W1	X	X
DECR2	ENSG00000242612	Q9NUI1	X	X
DHODH	ENSG00000102967	Q02127	X	X
DNAJC22	ENSG00000178401	Q8N4W6	X	X
DNAJC25	ENSG00000059769	Q9H1X3	X	X
ENSG00000273047	ENSG00000273047		No	No
ENSG00000273171	ENSG00000273171		No	No
ENSG00000289697	ENSG00000289697		No	No
EPO	ENSG00000130427	P01588	X	X
EVA1A	ENSG00000115363	Q9H8M9	X	X
F11	ENSG00000088926	P03951	X	X
F12	ENSG00000131187	P00748	X	X
F13B	ENSG00000143278	P05160	X	X
F2	ENSG00000180210	P00734	X	X
F7	ENSG00000057593	P08709	X	X
F9	ENSG00000101981	P00740	X	X
FCN2	ENSG00000160339	Q15485	No	No
FETUB	ENSG00000090512	Q9UGM5	X	X
FGA	ENSG00000171560	P02671	X	X
FGB	ENSG00000171564	P02675	X	X
FGF21	ENSG00000105550	Q9NSA1	X	X
FGG	ENSG00000171557	P02679	X	X
FGL1	ENSG00000104760	Q08830	X	X
FMO3	ENSG00000007933	P31513	X	X
FMO5	ENSG00000131781	P49326	X	X
FTCD	ENSG00000160282	O95954	X	X
FUOM	ENSG00000148803	A2VDF0	X	X
GBP7	ENSG00000213512	Q8N8V2	X	X
GC	ENSG00000145321	P02774	X	X

GCHFR	ENSG00000137880	P30047	X	X
GCKR	ENSG00000084734		X	X
GDF2	ENSG00000263761	Q9UK05	No	No
GGCX	ENSG00000115486	P38435	X	X
GLDC	ENSG00000178445	P23378	X	X
GLS2	ENSG00000135423	Q9UI32	X	X
GLTPD2	ENSG00000182327	A6NH11	X	X
GLYCTK	ENSG00000168237	Q8IVS8	X	X
GOLT1A	ENSG00000174567	Q6ZVE7	X	X
GPLD1	ENSG00000112293	P80108	X	X
GSTZ1	ENSG00000100577	O43708	X	X
GYS2	ENSG00000111713	P54840	No	No
HAAO	ENSG00000162882	P46952	X	X
HABP2	ENSG00000148702	Q14520	X	X
HAMP	ENSG00000105697	P81172	X	No
HAO1	ENSG00000101323	Q9UJM8	X	X
HGFAC	ENSG00000109758	Q04756	X	X
HMGCS2	ENSG00000134240	P54868	X	X
HP	ENSG00000257017	P00738	X	X
HPD	ENSG00000158104	P32754	X	X
HPR	ENSG00000261701	P00739	X	X
HPX	ENSG00000110169	P02790	X	X
HRG	ENSG00000113905	P04196	X	X
HSD11B1	ENSG00000117594	P28845	No	No
HSD17B13	ENSG00000170509	Q7Z5P4	X	X
HSD17B6	ENSG00000025423	O14756	X	X
HSD3B7	ENSG00000099377	Q9H2F3	X	X
IGFALS	ENSG00000099769	P35858	X	X
IGFBP1	ENSG00000146678	P08833	X	X
IL1RAP	ENSG00000196083	Q9NPH3	X	X
IL27	ENSG00000197272	Q8NEV9	X	X
INHBC	ENSG00000175189	P55103	X	X
INHBE	ENSG00000139269	P58166	X	X
INSIG1	ENSG00000186480	O15503	X	X
ITIH1	ENSG00000055957	P19827	X	X
ITIH2	ENSG00000151655	P19823	X	X
ITIH3	ENSG00000162267	Q06033	X	X
ITIH4	ENSG00000055955	Q14624	X	X
KDM8	ENSG00000155666	Q8N371	X	X
KLKB1	ENSG00000164344	P03952	X	X
KNG1	ENSG00000113889	P01042	X	X
LBP	ENSG00000129988	P18428	X	X
LEAP2	ENSG00000164406	Q969E1	X	X
LECT2	ENSG00000145826	O14960	X	No
LEPR	ENSG00000116678	P48357	X	X
LIME1	ENSG00000203896	Q9H400	X	X
LIPC	ENSG00000166035	P11150	X	X
LPA	ENSG00000198670	P08519	X	X
LRG1	ENSG00000171236	P02750	X	X
MASP2	ENSG00000009724	O00187	X	X
MAT1A	ENSG00000151224	Q00266	X	X

MBL2	ENSG00000165471	P11226	X	X
MLXIPL	ENSG00000009950	Q9NP71	X	X
MST1	ENSG00000173531		X	X
MT1B	ENSG00000169688	P07438	X	X
MTHFD1	ENSG00000100714	P11586	X	X
MTHFS	ENSG00000136371	P49914	X	X
NADK2	ENSG00000152620	Q4G0N4	X	X
NEU4	ENSG00000204099	Q8WWR8	X	X
NR1I3	ENSG00000143257	Q14994	X	X
OAF	ENSG00000184232	Q86UD1	X	X
OIT3	ENSG00000138315	Q8WWZ8	X	X
ORM1	ENSG00000229314	P02763	X	X
ORM2	ENSG00000228278	P19652	X	X
OSGIN1	ENSG00000140961	Q9UJX0	X	X
OXER1	ENSG00000162881	Q8TDS5	X	X
PCSK9	ENSG00000169174	Q8NBP7	X	X
PECR	ENSG00000115425	Q9BY49	X	X
PGLYRP2	ENSG00000161031	Q96PD5	X	X
PLG	ENSG00000122194	P00747	X	X
PLGLB2	ENSG00000125551	Q02325	X	X
PNPLA3	ENSG00000100344	Q9NST1	X	X
PON1	ENSG00000005421	P27169	X	X
PON3	ENSG00000105852	Q15166	X	X
PRAMEF10	ENSG00000187545	O60809	No	No
PRAMEF33	ENSG00000237700	A0A0G2JMD5	No	No
PROC	ENSG00000115718	P04070	X	X
PROX1	ENSG00000117707	Q92786	X	X
PROZ	ENSG00000126231	P22891	X	X
PZP	ENSG00000126838	P20742	X	X
RBP4	ENSG00000138207	P02753	X	X
RDH16	ENSG00000139547	O75452	X	X
RNASE4	ENSG00000258818	P34096	X	X
RTP3	ENSG00000163825	Q9BQQ7	X	No
SAA1	ENSG00000173432	P0DJI8	X	X
SAA2	ENSG00000134339	P0DJI9	X	X
SAA2-SAA4	ENSG00000255071		No	No
SAA4	ENSG00000148965	P35542	X	X
SDS	ENSG00000135094	P20132	X	X
SERPINA1	ENSG00000197249	P01009	X	X
SERPINA10	ENSG00000140093	Q9UK55	X	X
SERPINA11	ENSG00000186910	Q86U17	X	X
SERPINA4	ENSG00000100665	P29622	X	X
SERPINA6	ENSG00000170099	P08185	X	X
SERPINA7	ENSG00000123561	P05543	X	X
SERPINC1	ENSG00000117601	P01008	X	X
SERPIND1	ENSG00000099937	P05546	X	X
SERPINF2	ENSG00000167711	P08697	X	X
SHBG	ENSG00000129214	P04278	X	X
SLC10A1	ENSG00000100652	Q14973	X	X
SLC13A5	ENSG00000141485	Q86YT5	X	X
SLC17A2	ENSG00000112337	O00624	X	X

SLC22A1	ENSG00000175003	O15245	X	X
SLC22A10	ENSG00000184999	Q63ZE4	X	X
SLC22A25	ENSG00000196600	Q6T423	X	X
SLC22A7	ENSG00000137204	Q9Y694	X	X
SLC22A9	ENSG00000149742	Q8IVM8	X	X
SLC25A13	ENSG00000004864	Q9UJS0	X	X
SLC25A47	ENSG00000140107	Q6Q0C1	X	X
SLC27A5	ENSG00000083807	Q9Y2P5	X	X
SLC2A2	ENSG00000163581	P11168	X	X
SLC38A4	ENSG00000139209	Q969I6	X	X
SLCO1B1	ENSG00000134538	Q9Y6L6	X	X
SLCO1B3	ENSG00000111700	Q9NPD5	X	X
SMLR1	ENSG00000256162	H3BR10	X	X
SPP2	ENSG00000072080	Q13103	X	X
SULT2A1	ENSG00000105398	Q06520	X	X
TAT	ENSG00000198650	P17735	X	X
TDO2	ENSG00000151790	P48775	X	X
TFR2	ENSG00000106327	Q9UP52	X	X
THPO	ENSG00000090534	P40225	X	X
TLCD4	ENSG00000152078	Q96MV1	X	X
TMEM176B	ENSG00000106565	Q3YBM2	X	X
TMPRSS6	ENSG00000187045	Q8IU80	X	X
TTC36	ENSG00000172425	A6NLP5	X	X
TTPA	ENSG00000137561	P49638	X	X
UGT1A3	ENSG00000288702	P35503	X	X
UGT1A4	ENSG00000244474	P22310	X	X
UGT2B10	ENSG00000109181	P36537	X	X
UGT2B4	ENSG00000156096	P06133	X	X
UPB1	ENSG00000100024	Q9UBR1	X	X
UROC1	ENSG00000159650	Q96N76	X	No
VTN	ENSG00000109072	P04004	X	X
ZGPAT	ENSG00000197114	Q8N5A5	X	X

Table S2: Expression of 263 liver-specific, protein-coding genes according to the Human Protein Atlas and RNA-seq data (GSE288204) *in HuH7-NTCP vs HuH7.5-NTCP cells*

Supplementary references

Author names in bold designate shared co-first authorship

[1] Alfaiate D, Lucifora J, Abeywickrama-Samarakoon N et al. HDV RNA replication is associated with HBV repression and interferon-stimulated genes induction in super-infected hepatocytes. Antiviral Res 2016;136:19-31.

[2] Gripon P, Rumin S, Urban S et al. Infection of a human hepatoma cell line by hepatitis B virus. Proc Natl Acad Sci U S A 2002;99:15655-15660.

[3] Lecluyse EL, Alexandre E. Isolation and culture of primary hepatocytes from resected human liver tissue. Methods Mol Biol 2010;640:57-82.

[4] Ladner SK, Otto MJ, Barker CS et al. Inducible expression of human hepatitis B virus (HBV) in stably transfected hepatoblastoma cells: a novel system for screening potential inhibitors of HBV replication. Antimicrob Agents Chemother 1997;41:1715-1720.

[5] **Bach C, Lucifora J**, Delphin M et al. A stable hepatitis D virus-producing cell line for host target and drug discovery. Antiviral Res 2023;209:105477.

[6] Delgrange D, Pillez A, Castelain S et al. Robust production of infectious viral particles in Huh-7 cells by introducing mutations in hepatitis C virus structural proteins. J Gen Virol 2007;88:2495-2503.

[7] Pellerin M, Hirchaud E, Blanchard Y et al. Characterization of a Cell Culture System of Persistent Hepatitis E Virus Infection in the Human HepaRG Hepatic Cell Line. Viruses 2021;13.

[8] **Todt D, Friesland M**, Moeller N et al. Robust hepatitis E virus infection and transcriptional response in human hepatocytes. Proc Natl Acad Sci U S A 2020;117:1731-1741.

[9] Nielsen SU, Bassendine MF, Martin C et al. Characterization of hepatitis C RNA-containing particles from human liver by density and size. J Gen Virol 2008;89:2507-2517.

[10] Sayed IM, Verhoye L, Cocquerel L et al. Study of hepatitis E virus infection of genotype 1 and 3 in mice with humanised liver. Gut 2017;66:920-929.

[11] Peck D, Crawford ED, Ross KN et al. A method for high-throughput gene expression signature analysis. Genome Biol 2006;7:R61.

[12] Uhlen M, Fagerberg L, Hallstrom BM et al. Proteomics. Tissue-based map of the human proteome. Science 2015;347:1260419.

[13] Taverniti V, Meiss-Heydmann L, Gadenne C et al. CAM-A-dependent HBV core aggregation induces apoptosis through ANXA1. JHEP Rep 2024;6:101134.
